# Supplementary figures and images for: Nitrogen fertilizer application rate impacts eating and cooking quality of rice after storage
Source: PLoS One. 2021 Jun 18;16(6):e0253189. doi: 10.1371/journal.pone.0253189 (PMC8213157; doi:10.1371/journal.pone.0253189)

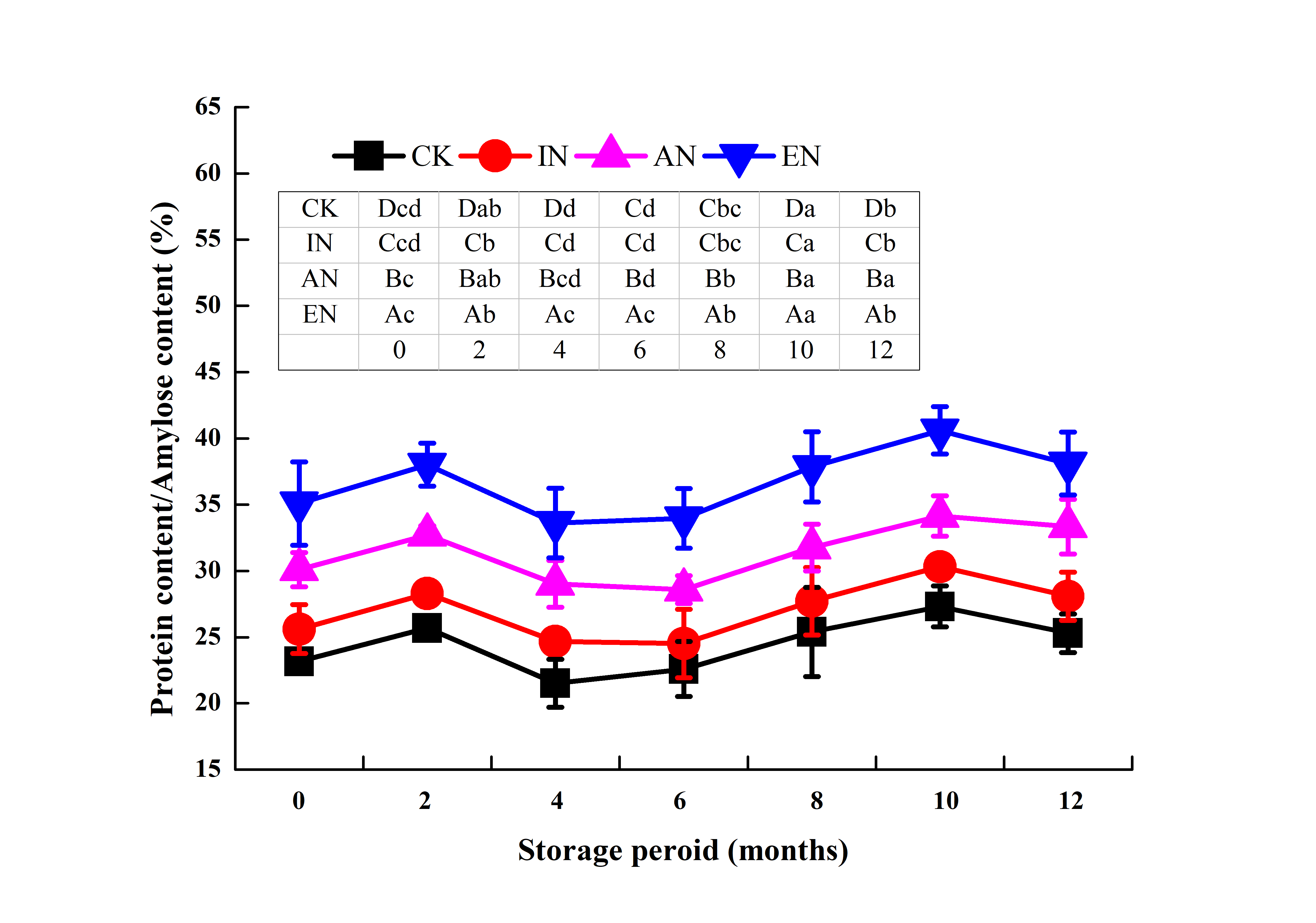

Supplement: S1 Fig — CK, control group (0 kg N/ha); IN, insufficient nitrogen (160 kg N/ha); AN, adequate nitrogen (260 kg N/ha); EN, excessive nitrogen (420 kg N/ha). Data (mean ± standard deviation, n = 9) with different letters are significantly different (p < 0.05). For each parameter, different lowercase letters in the same rows differ significantly as a function of storage time. Different uppercase letters in the column denote significant differences as a function of different nitrogen application rates. (TIF) [file pone.0253189.s001.tif]
