# Supplementary material for: Nitrogen fertilizer application rate impacts eating and cooking quality of rice after storage
Source: PLoS One. 2021 Jun 18;16(6):e0253189. doi: 10.1371/journal.pone.0253189 (PMC8213157; doi:10.1371/journal.pone.0253189)
Supplement: S1 Table — (DOCX) [file pone.0253189.s002.docx]

**S1 Table. Significance of variance estimates related to the interactions between storage time and nitrogen application rates on the quality traits of rice grain.**

| **Eating and cooking quality** | **Nitrogen × Storage time** |
| --- | --- |
| **Chemical components** |  |
| Amylose content | ns |
| Protein content | ns |
| Fat content | ns |
| Moisture | ns |
| **Eating quality** |  |
| Taste value | ns |
| Hardness | ns |
| Gumminess | * |
| Springiness | ns |
| **Cooking quality** |  |
| Cooking time | ns |
| Water uptake | ns |
| Volume expansion | ns |
| Rice soup pH | ns |
| **Pasting characteristics** |  |
| Peak viscosity | ns |
| Trough viscosity | * |
| Breakdown | ns |
| Final viscosity | ns |
| Setback | * |
| Peak time | ns |
| Pasting temperature | ns |

ns, not significant; *, significance at *P* ≤ 0.05
