# Supplementary material for: Nitrogen fertilizer application rate impacts eating and cooking quality of rice after storage
Source: PLoS One. 2021 Jun 18;16(6):e0253189. doi: 10.1371/journal.pone.0253189 (PMC8213157; doi:10.1371/journal.pone.0253189)
Supplement: Attachment — Submitted filename: PONE-D-21-06634_reviewer.pdf [file pone.0253189.s004.pdf]

PLOS ONE

Nitrogen fertilizer application rate impacts eating and cooking quality of rice after storage

--Manuscript Draft--

**Manuscript Number:**	PONE-D-21-06634
**Article Type:**	Research Article
**Full Title:**	Nitrogen fertilizer application rate impacts eating and cooking quality of rice after storage
**Short Title:**	Effect of nitrogen fertilization rate on rice quality post-storage
**Corresponding Author:**	Hanling Liang<br>Shenyang Agricultural University<br>Shenyang, CHINA
**Keywords:**	nitrogen; fertilizer; storage; rice; eating quality; cooking quality
**Abstract:**	The effect of nitrogen fertilizer application on the quality of rice during storage is not well understood. The eating and cooking quality (ECQ) of rice treated with 0 (CK, control), 160 (IN, insufficient nitrogen), 260 (AN, adequate nitrogen), and 420 (EN, excessive nitrogen) kg N/ha was analyzed over 12 months of storage. Results showed that the rate of nitrogen fertilizer application had no significant impact on the changes in taste value during storage. However, EN application significantly increased the hardness ($p < 0.05$) and reduced the gumminess of rice ($p < 0.05$), and delayed the decline in the viscosity of rice paste by two months after storage of one years. In conclusion, although EN application resulted in an inferior texture of rice, it delayed quality change by two months during storage. It was demonstrated that a rational nitrogen application rate (0–260 kg N/ha) for rice cultivation is particularly important to obtain high ECQ, but EN might be beneficial for the stability of the ECQ during storage.
**Order of Authors:**	Hanling Liang
	Dongbing Tao
	Qi Zhang
	Shuang Zhang
	Jiayi Wang
	Lifei Liu
	Zhaoxia Wu
	Wen Tao Sun
**Additional Information:**	
**Question**	**Response**
**Financial Disclosure**<br><br>Enter a financial disclosure statement that describes the sources of funding for the work included in this submission. Review the submission guidelines for detailed requirements. View published research articles from *PLOS ONE* for specific examples.<br><br>This statement is required for submission and **will appear in the published article** if the submission is accepted. Please make	This work was supported by grants from the National Key Research and Development Program of China (Grant No. 2018YFD0300300), and Science and Technology Innovation Talents Training Project of Liaoning Province (Grant No. XLYC1802044).

Powered by Editorial Manager® and ProduXion Manager® from Aries Systems Corporation

sure it is accurate.<br><br>**Unfunded studies**<br>Enter: *The author(s) received no specific funding for this work.*<br><br>**Funded studies**<br>Enter a statement with the following details:<br>• Initials of the authors who received each award<br>• Grant numbers awarded to each author<br>• The full name of each funder<br>• URL of each funder website<br>• Did the sponsors or funders play any role in the study design, data collection and analysis, decision to publish, or preparation of the manuscript?<br>• **NO** - Include this sentence at the end of your statement: *The funders had no role in study design, data collection and analysis, decision to publish, or preparation of the manuscript.*<br>• **YES** - Specify the role(s) played.<br><br>* typeset	
**Competing Interests**<br><br>Use the instructions below to enter a competing interest statement for this submission. On behalf of all authors, disclose any competing interests that could be perceived to bias this work—acknowledging all financial support and any other relevant financial or non-financial competing interests.<br><br>This statement is required for submission and **will appear in the published article** if the submission is accepted. Please make sure it is accurate and that any funding sources listed in your Funding Information later in the submission form are also declared in your Financial Disclosure statement.<br><br>View published research articles from *PLOS ONE* for specific examples.	The authors have declared that no competing interests exist.

**NO authors have competing interests**<br><br>Enter: *The authors have declared that no competing interests exist.*<br><br>**Authors with competing interests**<br><br>Enter competing interest details beginning with this statement:<br><br>*I have read the journal's policy and the authors of this manuscript have the following competing interests: [insert competing interests here]*<br><br>* typeset	
**Ethics Statement**<br><br>Enter an ethics statement for this submission. This statement is required if the study involved:<br><br>• Human participants<br>• Human specimens or tissue<br>• Vertebrate animals or cephalopods<br>• Vertebrate embryos or tissues<br>• Field research<br><br>Write "N/A" if the submission does not require an ethics statement.<br><br>General guidance is provided below. Consult the submission guidelines for detailed instructions. **Make sure that all information entered here is included in the Methods section of the manuscript.**	N/A

**Format for specific study types**<br><br>**Human Subject Research (involving human participants and/or tissue)**<br>• Give the name of the institutional review board or ethics committee that approved the study<br>• Include the approval number and/or a statement indicating approval of this research<br>• Indicate the form of consent obtained (written/oral) or the reason that consent was not obtained (e.g. the data were analyzed anonymously)<br><br>**Animal Research (involving vertebrate animals, embryos or tissues)**<br>• Provide the name of the Institutional Animal Care and Use Committee (IACUC) or other relevant ethics board that reviewed the study protocol, and indicate whether they approved this research or granted a formal waiver of ethical approval<br>• Include an approval number if one was obtained<br>• If the study involved *non-human primates*, add *additional details* about animal welfare and steps taken to ameliorate suffering<br>• If anesthesia, euthanasia, or any kind of animal sacrifice is part of the study, include briefly which substances and/or methods were applied<br><br>**Field Research**<br><br>Include the following details if this study involves the collection of plant, animal, or other materials from a natural setting:<br>• Field permit number<br>• Name of the institution or relevant body that granted permission	
**Data Availability**<br><br>Authors are required to make all data underlying the findings described fully available, without restriction, and from the time of publication. PLOS allows rare exceptions to address legal and ethical concerns. See the PLOS Data Policy and FAQ for detailed information.	Yes - all data are fully available without restriction

Powered by Editorial Manager® and ProduXion Manager® from Aries Systems Corporation

A Data Availability Statement describing where the data can be found is required at submission. Your answers to this question constitute the Data Availability Statement and **will be published in the article**, if accepted.<br><br>**Important:** Stating 'data available on request from the author' is not sufficient. If your data are only available upon request, select 'No' for the first question and explain your exceptional situation in the text box.<br><br>Do the authors confirm that all data underlying the findings described in their manuscript are fully available without restriction?	
**Describe where the data may be found in full sentences. If you are copying our sample text, replace any instances of XXX with the appropriate details.**<br><br>• If the data are **held or will be held in a public repository**, include URLs, accession numbers or DOIs. If this information will only be available after acceptance, indicate this by ticking the box below. For example: *All XXX files are available from the XXX database (accession number(s) XXX, XXX.*).<br>• If the data are all contained **within the manuscript and/or Supporting Information files**, enter the following: *All relevant data are within the manuscript and its Supporting Information files.*<br>• If neither of these applies but you are able to provide **details of access elsewhere**, with or without limitations, please do so. For example:<br><br>*Data cannot be shared publicly because of [XXX]. Data are available from the XXX Institutional Data Access / Ethics Committee (contact via XXX) for researchers who meet the criteria for access to confidential data.*<br><br>*The data underlying the results presented in the study are available from (include the name of the third party*	All relevant data are within the manuscript and its Supporting Information files.

and contact information or URL).<br>• This text is appropriate if the data are owned by a third party and authors do not have permission to share the data.<br><br>* typeset	
Additional data availability information:	

Powered by Editorial Manager® and ProduXion Manager® from Aries Systems Corporation

Manuscript Click here to access/download;Manuscript;main_article.docx

Nitrogen fertilizer application rate impacts eating and cooking quality of rice after storage

Short title: Effect of nitrogen fertilization rate on rice quality post-storage

Hanling Liang[1], Dongbing Tao[1], Qi Zhang[1], Shuang Zhang[1], Jiayi Wang[2], Lifei Liu[1], Zhaoxia Wu[1*], Wentao Sun[3*]

[1] College of Food Science, Shenyang Agricultural University, Shenyang, 110866, People's Republic of China

[2] College of Food and Chemical Engineering, Shaoyang University, Shaoyang, 422000, People's Republic of China

[3] Institute of Plant Nutrition and Environmental Resources, Liaoning Academy of Agricultural Sciences, Shenyang, 110161, People's Republic of China

*Corresponding author

E-mail: wuzxsau@163.com (ZW); wentaosw@163.com (WS)

Abstract

The effect of nitrogen fertilizer application on the quality of rice during storage is not well understood. The eating and cooking quality (ECQ) of rice treated with 0 (CK, control), 160 (IN, insufficient nitrogen), 260 (AN, adequate nitrogen), and 420 (EN, excessive nitrogen) kg N/ha was analyzed over 12 months of storage. Results showed that the rate of nitrogen fertilizer application had no significant impact on the changes in taste value during storage. However, EN application significantly increased the hardness ($p < 0.05$) and reduced the gumminess of rice ($p < 0.05$), and delayed the decline in the viscosity of rice paste by two months after storage of one years. In conclusion, although EN application resulted in an inferior texture of rice, it delayed quality change by two months during storage. It was demonstrated that a rational nitrogen application rate (0–260 kg N/ha) for rice cultivation is particularly important to obtain high ECQ, but EN might be beneficial for the stability of the ECQ during storage.

Keywords: nitrogen; fertilizer; storage; rice; eating quality; cooking quality

Introduction

Rice, as a staple food, plays an important role in human diet, and is usually stored for long periods to meet the needs of people owing to its seasonal growth. However, many studies have reported that the storage process may result in the deterioration of the eating quality of rice, including increased hardness and reduced viscosity (Anonymous, 2016a).

Furthermore, the cooking and gelatinization characteristics may be changed, including increase in water absorption, volume expansion rate, cooking time, and setback, as well as a decrease in tro[illegible] viscosity, final viscosity, and breakdown (Keawpeng and Venkatachalam, 2015). This deterioration is mostly considered to be related to changes in the interactions between the proteins and starch within the rice grain (Chrastil, 1990; Guo *et al.*, 2015; Huang and Lai, 2014; Patindol *et al.*, 2005; Zhou *et al.*, 2003).

Numerous efforts have been made in recent years to reduce the deterioration of rice quality due to storage. Low temperature and humidity (Genkawa *et al.*, 2008; Park *et al.*, 2012), as well as vacuum or nano packaging (Wang *et al.*, 2018) have proven to be beneficial in maintaining rice quality during storage. The methods have been applied in the storage of milled rice, which has a high commodity price, but not in rice paddy storage owing to the trade-off between the high cost of the storage method and the relatively low commodity value of rice paddy, which is also the reason why rice paddy is usually stored under natural conditions. To date, no feasible solution to the problem of quality loss during rice paddy storage is available. Except for storage conditions, the initial quality of rice is also a key factor for the end-use quality of rice after storage. Fertilization is important for the establishment of the initial quality of rice, especially the rate of nitrogen application rate. It is well-documented that nitrogen fertilizers have a prominent effect on the eating and cooking quality (ECQ) of rice (Kaur *et al.*, 2016), mainly owing to the increased protein content and decreased amylose content of rice (Cao *et al.*, 2017) resulting from nitrogen application, which causes an increase in the hardness of rice, decreasing its palatability

(Champagne *et al.*, 2009; Singh *et al.*, 2011; Zhu *et al.*, 2017b), and also alters the rice gelatinization characteristics and cooking quality.

The effect of nitrogen application rate on the initial quality of fresh rice is well known, but whether this effect persists during a period of storage is still unclear. Previous studies have indicated that fertilization has certain effects on the quality of tomato and potato during storage (Makani *et al.*, 2017; Unlu *et al.*, 2010). However, there are no reports addressing whether different nitrogen fertilization levels affect rice quality during storage. In this study, we explored the dynamic changes in the ECQ during storage of rice produced using different nitrogen application rates, and discussed the likely reasons behind the variations in ECQ after storage, with the objective of providing a theoretical basis for the production and quality-control of high-quality rice from the field to the table.

Material and methods

Materials

The experiment was conducted at Liaohe Delta, Panjin, China (122°14′17″N, 41°9′31″E). The physical and chemical properties of the 0–20 cm soil was as follows: pH 8.2, organic matter 22.57 g/kg, total nitrogen 1.42 g/kg, alkali nitrogen 105.24 mg/kg, available phosphorus 21.61 mg/kg, available potassium 164.22 mg/kg, and bulk density 1.39 g/cm^3. The following treatment groups were set up for our studies: a control (CK) group without nitrogen treatment (0 kg N/ha), and three treatment groups with insufficient (IN; 160 kg N/ha), adequate (AN; 260 kg N/ha), and excessive (EN; 420 kg N/ha) nitrogen, based on

the typical nitrogen fertilization of 260 kg N/ha used by local farmers. Except for the nitrogen application rates, standard rice cultivation procedures were followed as done by the local farmers. The rice cultivar was Yanfeng 47, which is one of the main local varieties. The seedlings were transplanted on May 25, and harvested on October 8 in 2018.

Sample preparation

The grains were air-dried for a month to reduce moisture, and then stored under laboratory conditions for 12 months. The temperature and humidity conditions in the laboratory were recorded every three days, as shown in Fig 1. The paddy rice (500 g) was processed at an interval of two months in a ridge mill (FC2K, Yamamoto, Japan) and a milling machine (VP-32T, Yamamoto, Japan) to obtain brown and milled rice, respectively, for subsequent analyses.

Fig 1. Record of temperature and relative humidity in the laboratory during the experimental period from November 2018 to November 2019.

Chemical component analysis

The protein and amylose contents of brown rice were measured with a near-infrared grating nutrient analyzer (DA7200, Perten, Sweden). Fat content was determined with the Soxhlet extraction method described in GB 5009.6-2016 (Anonymous, 2016b). Rice moisture was determined using the method described in GB 5009.3-2016 (Anonymous, 2016a).

Eating quality analysis

The taste value of the cooked rice was assessed according to Champagne et al. (Champagne *et al.*, 1996). Briefly, 30 g of milled rice was cooked according to GB/T 15682-2008 (Anonymous, 2008), and then 8 g of the cooked rice was pressed into rice cakes, and the taste value was measured with a rice taste analyzer (STA1B, Satake, Japan). The texture of the rice cakes (hardness, gumminess, and springiness, defined for instrumental texture analyses in the study by Champagne et al. (Champagne *et al.*, 1999)) was determined with the Texture Analyzer (Brookfield Engineering Laboratories, MA, US) according to Zhang et al. (Zhang *et al.*, 2019). The sample was compressed using a 35 mm global probe attachment at a speed of 2 mm/second. The texture profile analysis settings were as follows: pre-test speed, 2.00 mm/s; post-test speed, 2.00 mm/s; time, 10 s; trigger force, 0.05 N. The measurement was conducted in ten replicates.

Cooking quality analysis

The cooking quality was determined following the method of Gujral and Kumar (Gujral and Kumar, 2003), with minor modifications. Briefly, 2.5 grams of milled rice was boiled at 100 °C for 10 min in a beaker containing 50 mL distilled water. Then, three grains of rice were removed every minute, and pressed between two glass slides until the grains showed no chalky core; the total time was recorded as the cooking time and the measurement was done in three replicates. For volume expansion and water uptake, we measured the volume and weighed 2.5 g of milled rice before and after boiling, according to the aforementioned method for the cooking time. The pH of rice soup was determined

with a pH meter (PB-10, Sartorius, Germany). Volume expansion and water uptake were calculated with the following formulae:

$$\text{Volume expansion (\%)} = \frac{\text{Volume of cooked rice} - \text{Volume of uncooked rice}}{\text{Volume of uncooked rice}} \times 100$$

$$\text{Water uptake (\%)} = \frac{\text{Weight of cooked rice} - \text{Weight of uncooked rice}}{\text{Weight of uncooked rice}} \times 100$$

Pasting characteristic analysis

The pasting parameters of milled rice flour, such as peak viscosity, trough viscosity, breakdown, final viscosity, setback, peak time, and pasting temperature (Fig 2), were determined with a rapid viscosity analyzer (RVA-4, Newport Scientific, Australia). Milled rice flour (3.0 mg) was passed through a 100-mesh screen into a sample box containing 25 mL distilled water, and then analyzed with the rapid viscosity analyzer.

Fig 2. Rapid viscosity analyzer profile of milled rice flour.

Statistical analysis

All data were processed using Microsoft Excel 2019, and then analyzed for significance using SPSS 22 software with the Duncan's test ($p < 0.05$). All tables were generated with Microsoft Word 2019. Graphs were plotted using Origin 8.5. Analysis of variance and linear correlation analysis were conducted using SPSS 22, and linear regression was used for Pearson correlation analysis between rice quality and chemical components during storage.

Results and discussion

ECQ and chemical composition of rice

Among the ECQ indices evaluated, nitrogen application rates and storage time only showed significant interaction effects on gumminess, trough viscosity, and setback, ($p < 0.05$; data not shown), indicating that nitrogen application rate has a significant impact on the ECQ of rice during postharvest storage. Thus, controlling the nitrogen application rate in the fields is important for maintaining the ECQ of rice during storage.

Content of chemical components

Nitrogen application did not impact the change of protein, fat, and moisture contents in rice during storage (Fig 3). Amylose content in the CK and IN samples did not vary significantly during storage, but EN treatment significantly exacerbated the rate of amylose decline during the 4th to 10th months of storage, which demonstrated that nitrogen application in the field changed the starch metabolism of rice during storage.

During the storage period of 4–10 months, the amylose content decreased by 1% in the CK samples, and 4% with EN treatment. The decline in amylose content of rice during storage was also observed in the study by Gu et al., and was attributed to endogenous degradation (Gu *et al.*, 2019). α-Amylase is the main enzyme involved in starch hydrolysis. It has been reported that the activity of α-amylase in rice is higher under higher nitrogen application rates (Zhu *et al.*, 2017a). Therefore, for our study, it is possible that nitrogen

application increased the activity of α-amylase in rice, and accelerated the degradation of amylose during storage. However, this effect seems to be dependent on nitrogen application rate only in the case of EN, which is significant ($p < 0.05$). Moreover, since amylose content is closely related to the ECQ of rice (Derycke *et al.*, 2005; Li *et al.*, 2016), the variations in amylose content between the different nitrogen application rates may have contributed to the differential changes in rice ECQ during storage.

Fig 3. The changes in the content of chemical components in rice under storage, following the application of nitrogen at different rates. CK, control group (0 kg N/ha); IN, insufficient nitrogen (160 kg N/ha); AN, adequate nitrogen (260 kg N/hm^2); EN, excessive nitrogen (420 kg N/ha). Data (mean ± standard deviation, n = 9) with different letters are significantly different ($p < 0.05$). For each parameter in the inset table, different lowercase letters in the same column differ significantly as a function of storage time as per Duncan's test ($p< 0.05$). Different uppercase letters in the rows denote significant differences as a function of nitrogen application rates as per Duncan's test ($p < 0.05$).

Eating quality

Taste value

As shown in Fig 4, rice exposed to different nitrogen application rates showed similar variations in taste value during storage. The taste value of rice was significantly reduced with a higher nitrogen application rate, and slightly decreased after storage, which is consistent with previous studies (Liu *et al.*, 2002; Zhu *et al.*, 2017b). Besides, without

storage, the taste value of rice under EN treatment decreased from 63 to 48 than CK group. For CK group, the taste value of rice stored for 12 months treatments decreased from 63 to 60 than without storage (Fig 4). Notably, nitrogen application had a greater impact on the taste value of rice than storage under the conditions of this study. Accordingly, controlling the rate of nitrogen application for cultivating rice is particularly important for maintaining the high eating quality of rice during storage.

Textural characteristics

Hardness, gumminess, and springiness are important parameters for the textural evaluation of cooked rice (Zhou *et al.*, 2007). In this study, nitrogen application increased the hardness, and reduced the gumminess, but had little impact on the springiness of cooked rice, as compared before and after storage (Fig 4), which means that nitrogen application accelerated the degree of deterioration of textural characteristics of rice during storage.

As shown in Fig 4, the change in hardness of rice after storage for 12 months for the CK, IN, AN, and EN groups was -11%, +16%, +11%, and +14%, respectively, compared to rice without storage. Further, change in gumminess of rice from the CK, IN, AN, and EN groups was -40%, +16%, -47%, and -48%, respectively.

Hardness is positively related to the amylose content (Li *et al.*, 2016), and gumminess is closely related to the amylopectin content in the leachate produced by cooking the rice (Li *et al.*, 2019), especially the high proportion of short chain amylopectin (Li *et al.*, 2016). However, in our study, the more profound increase in hardness and decline in gumminess

for 0–12 months of storage under EN treatments seemed to be somewhat linked to the changes in the chemical components alone (protein, amylose, fat, and moisture contents), as shown in Fig 3. Moreover, the increase in hardness and decline in gumminess during storage is mainly due to the formation of amylose–lipid complexes (Sodhi *et al.*, 2007) and the increase in the content of high molecular weight proteins caused by protein aggregation (Tulyathan and Leeharatanaluk, 2007). Further, the solubility of protein and amylose is reduced owing to changes in their interactions in rice, and results in reduced gumminess (Zhou *et al.*, 2002). However, the decreased amylose content observed in our study may not be conducive to the formation of amylose–lipid complexes. It has been proven that nitrogen application increases the high molecular weight subunits in protein fractions (Kaur *et al.*, 2016). Thus, in this experiment, it is possible that the nitrogen application had a greater effect on the increase in the content of high molecular weight proteins during storage (especially for late-storage), which caused marked reduction in the solubility of rice components, and resulted in increased hardness and reduced gumminess after storage. However, further investigations are needed to verify this observation.

Fig 4. The changes in the eating quality of rice during storage, following the application of nitrogen at different rates. CK, control group (0 kg N/ha); IN, insufficient nitrogen (160 kg N/ha); AN, adequate nitrogen (260 kg N/ha); EN, excessive nitrogen (420 kg N/ha). For each parameter, the mean ± standard deviation (n = 3) followed by different lowercase letters in the same column differ significantly as per Duncan's test ($p < 0.05$) as a function of storage time. Different uppercase letters in the rows denote significant

differences as per Duncan's test ($p < 0.05$) as a function of different nitrogen application rates.

Cooking quality

Except for the pH of the rice soup, there were no significant differences in the changes in cooking quality index during storage between different nitrogen application rates (Fig 5), which means that nitrogen fertilizer application during cultivation had little impact on the change in cooking quality of rice during storage.

Compared with other treatments, EN treatment exacerbated the reduction in the pH of rice soup during storage for 4–6 months. The changes in the pH of rice soup for CK, IN, AN, and EN groups were -0.7%, +0.7%, -0.3%, and -3%, respectively. Further, the EN treatment maintained a low pH of rice soup even after storage of rice for 6 months.

The principal sources of acidic substances in rice during storage are the free fatty acids produced during fat oxidation (Zhou *et al.*, 2002). EN may have increased the activity of enzymes related to lipid oxidation, such as lipoxygenase, and increased lipid oxidation to produce more fatty acids, resulting in a greater reduction in the pH of rice soup than the CK group. Our results confirmed that the fatty acid concentration of rice for the EN treatment was higher than that of the other treatments at late storage (data not shown). Although the change in pH of the rice soup was not enough to cause sensory differences, it may be possible that the changes in fatty acids, flavor substances, and other metabolites might be due to the change in pH, and deserves further investigation.

Fig 5. Changes in the cooking quality of rice during storage, following the application of nitrogen at different rates. CK, control group (0 kg N/ha); IN, insufficient nitrogen (160 kg N/ha); AN, adequate nitrogen (260 kg N/ha); EN, excessive nitrogen (420 kg N/ha). For each parameter, the mean ± standard deviation (n = 3) followed by different lowercase letters in the same column differ significantly as per Duncan's test ($p < 0.05$) as a function of storage time. Different uppercase letters in the rows denote significant differences as per Duncan's test ($p < 0.05$) as a function of different nitrogen application rates.

Pasting characteristics of rice flour

Pasting characteristics of rice flour are considered to be closely related to the ECQ of rice (Fitzgerald *et al.*, 2003), and are also one of the sensitive indicators that reflect rice aging during storage (Keawpeng and Venkatachalam, 2015). The rate of decline in viscosity index usually represents the aging of rice.

As shown in Fig 6, small differences were observed between the four nitrogen application rates in terms of the changes in setback, breakdown, peak time, and pasting temperature during storage. Further, the viscosity indices (peak viscosity, final viscosity, and trough viscosity) of rice in the AN and EN treatments were lower than that in CK during the one-year storage period, which indicated that AN and EN application were not conducive to maintaining good viscosity of rice, which is consistent with a previous study (Yang *et al.*, 2016). Nevertheless, the decline of trough viscosity started in rice after four months of storage for the CK and IN groups, and after six months for the AN and EN

treatments. For final viscosity, the trend was similar to that of the trough viscosity. It implies that AN and EN application delayed the aging of rice during storage to some extent.

Although this paper only discusses the change in pasting characteristics over one year, considering that the storage time of rice in actual production may be far more than one year, it can be imagined that if the storage is prolonged, this effect due to the delay may be more obvious. Therefore, this observation may be of great significance in delaying the change of pasting characteristics during rice aging in the future, and the underlying mechanisms are worth further study.

Fig 6. The changes in pasting characteristics of rice during storage, following nitrogen application at different rates. CK, control group (0 kg N/ha); IN, insufficient nitrogen (160 kg N/ha); AN, adequate nitrogen (260 kg N/ha); EN, excessive nitrogen (420 kg N/ha). Data (mean ± standard deviation, n = 9) with different letters are significantly different ($p < 0.05$). For each parameter, different lowercase letters in the same column differ significantly as a function of storage time. Different uppercase letters in the rows denote significant differences as a function of different nitrogen application rates.

Correlation between rice quality index and changes in chemical composition

In all the experimental groups, the effect of protein/amylose ratio on the ECQ of rice was greater than that of the amylose and protein contents alone (Table 1), which was a complement to what is usually observed upon analyzing amylose and protein contents and their correlation with ECQ of rice (Kim *et al.*, 2010; Ramesh *et al.*, 2000). We observed that

the protein/amylose ratio had a greater impact on the eating quality of rice during storage, implying that greater emphasis must be placed on this index when considering the changes in rice quality during future storage operations. Besides, the protein/amylose ratio had a significantly ($p<0.05$) negative correlation with breakdown, and the peak and trough viscosity (Table 1). Apparently, the change in peak and trough viscosity of rice, delayed by excessive nitrogen application (Fig 4), may be related to the change in protein/amylose ratio. In this study, this ratio was at a high level in the EN treatment (data not shown), which means that high protein/amylose ratio may be beneficial in the stability of rice. Nonetheless, the observation is worth further investigation and validation in other rice varieties.

Table 1. Correlation analysis between taste quality indices and chemical composition changes of rice during storage, following nitrogen application at different rates.

	Protein content				Amylose content				Protein content/amylose content			
	**N0**	**N160**	**N260**	**N420**	**N0**	**N160**	**N260**	**N420**	**N0**	**N160**	**N260**	**N420**
**Taste value**	-.893**								-.848*	-.848*	-.848*	-.848*
**Hardness**						-.893**						
**Gumminess**		-.786*					.786*					
**Springiness**				-.775*			.964**		-.818*	-.818*	-.818*	-.818*
**Cooking time**				-.757*	.811*				-.798*	-.798*	-.798*	-.798*
**Water uptake**				-.883**	.893**				-.815*	-.815*	-.815*	-.815*
**Volume expansion**		-.821*		-.955**								
**Rice soup pH**		.786*		.883**								
**Peak viscosity**										-.891**	-.820*	-.805*
**Trough viscosity**										-.855*	-.904**	-.886**
**Breakdown**										-.855*	-.907**	-.888**
**Setback**										-.777*		
**Peak time**			-.821*	-.818*						-.794*		-.776*

*p < 0.05, **p < 0.01.

317

Conclusions

To our knowledge, this is the first report on the effect of nitrogen fertilizer levels on rice quality during storage. Results showed that excessive nitrogen application significantly increased the hardness and reduced the gumminess during late storage. AN and EN applications delayed the change in the viscosity of rice paste. In addition, during storage, the protein/amylose ratio showed a better correlation to the ECQ of rice than the protein or amylose contents alone. The results demonstrated that fertilizer application rate affected the quality of the agricultural product during storage by influencing the initial quality of the raw material. Hence, more attention should be paid to the effects of pre-harvest field fertilization on the quality changes of agricultural products during storage, so as to better ensure the high-quality of agricultural products from the field to the plate.

Acknowledgements

Hanling Liang contributed to the conception and data analyses of the study and wrote the manuscript. Zhaoxia Wu and Wentao Sun supervised the entire project and contributed significantly to manuscript preparation. Dongbing Tao, Qi Zhang, Shuang Zhang, Jiayi Wang, and Lifei Liu determined the postharvest quality of rice. All authors read and approved the final manuscript.

The authors appreciate the contributions of Tian Zhang, Chen Yang, Yubo Liu, Wanning Zhao, Yu Fu, Tianyu Wang, Liyan Rong, and Baiyu Gu in the experiments.

Funding

This work was supported by grants from the National Key Research and Development Program of China (Grant No. 2018YFD0300300), and Science and Technology Innovation Talents Training Project of Liaoning Province (Grant No. XLYC1802044).

REFERENCES

Anonymous, 2008. Grain and oil inspection, Sensory evaluation method for cooking quality of rice and rice. General Administration of Quality Supervision Inspection and Quarantine of China.

Anonymous, 2016a. National food safety standards, Determination of moisture in food. National Health and Family Planning Commission of China.

Anonymous, 2016b. National food safety standards, Determination of fat in food. State Food and drug administration of China.

Cao, X.M., Sun, H.Y., Wang, C.G., Ren, X.J., Liu, H.F. and Zhang, Z.J., 2017. Effects of late-stage nitrogen fertilizer application on the starch structure and cooking quality of rice. Journal of the Science of Food and Agriculture 98: 2332-2340. 10.1002/jsfa.8723

Champagne, E.T., Bett-Garber, K.L., Thomson, J.L. and Fitzgerald, M.A., 2009. Unraveling the impact of nitrogen nutrition on cooked rice flavor and texture. Cereal Chemistry 86: 274-280. 10.1094/CCHEM-86-3-0274

Champagne, E.T., Bett, K.L., Vinyard, B.T., McClung, A.M., Barton, F.E., Moldenhauer, K., Linscombe, S. and McKenzie, K., 1999. Correlation between cooked rice texture and rapid visco analyser measurements. Cereal Chemistry 76: 764-771.

Champagne, E.T., Richard, O.A., Bett, K., Grimm, C., Vinyard, B., Webb, B., McClung, A., Barton, F., Lyon, B. and Moldenhauer, K., 1996. Quality evaluation of US medium-grain rice using a Japanese taste analyzer. Cereal Chemistry 73: 290-294.

Chrastil, J., 1990. Protein-starch interactions in rice grains. Influence of storage on oryzenin and starch. Journal of Agricultural and Food Chemistry 38: 1804-1809.

Derycke, V., Veraverbeke, W.S., Vandeputte, G.E., Man, W.D., Hoseney, R.C. and Delcour, J.A., 2005. Impact of Proteins on Pasting and Cooking Properties of Nonparboiled and Parboiled Rice. Cereal Chemistry 82: 468-474.

Fitzgerald, M.A., Martin, M., Ward, R.M., Park, W.D. and Shead, H.J., 2003. Viscosity of rice flour: a rheological and biological study. Journal of Agricultural and Food Chemistry 51: 2295-2299. 10.1021/jf020574i

Genkawa, T., Uchino, T., Inoue, A., Tanaka, F. and Hamanaka, D., 2008. Development of a low-moisture-content storage system for brown rice: Storability at decreased moisture contents. Biosystems Engineering 99: 515-522.

Gu, F., Gong, B., Gilbert, R.G., Yu, W., Li, E. and Li, C., 2019. Relations between changes in starch molecular fine structure and in thermal properties during rice grain storage. Food Chemistry 295: 484-492.

Gujral, H.S. and Kumar, V., 2003. Effect of accelerated aging on the physicochemical and textural properties of brown and milled rice. Journal of Food Engineering 59: 117-121.

Guo, Y.B., Cai, W.R., Tu, K., Wang, S.M. and Zhu, X.L., 2015. Key Proteins Causing Changes in Pasting Properties of Rice During Aging. Cereal Chemistry 92: 384-388. 10.1094/cchem-05-14-0104-r

Huang, Y.-C. and Lai, H.-M., 2014. Characteristics of the starch fine structure and pasting properties of waxy rice during storage. Food Chemistry 152: 432-439. 10.1016/j.foodchem.2013.11.144

Kaur, A., Ghumman, A., Singh, N., Kaur, S., Virdi, A.S., Riar, G.S. and Mahajan, G., 2016. Effect of different doses of nitrogen on protein profiling, pasting and quality attributes of rice from different cultivars. J Food Sci Technol 53: 2452-2462. 10.1007/s13197-016-2230-z

Keawpeng, I. and Venkatachalam, K., 2015. Effect of aging on changes in rice physical qualities. International Food Research Journal 22: 2180-2187.

Kim, Y.J., Choi, S.H., Han, Y.J., Jang, I.C., Song, J.T., Bhoo, S.H. and Seo, H.S., 2010. Rice Protein Disulfide Isomerase (OsPDI) and Rice Chaperone Binding Protein (OsBiP) Levels Are Not Directly Correlated with the Eating Quality of Rice. Journal of the Korean Society for Applied Biological Chemistry 53: 508-511. 10.3839/jksabc.2010.077

Li, H.Y., Lei, N.Y., Yan, S., Gao, M.Y., Yang, J.Y., Wang, J. and Sun, B.G., 2019. Molecular causes for the effect of cooking methods on rice stickiness: A mechanism explanation from the view of starch leaching. International Journal

of Biological Macromolecules 128: 49-53. 10.1016/j.ijbiomac.2019.01.113

Li, H.Y., Prakash, S., Nicholson, T.M., Fitzgerald, M.A. and Gilbert, R.G., 2016. The importance of amylose and amylopectin fine structure for textural properties of cooked rice grains. Food Chemistry 196: 702-711. 10.1016/j.foodchem.2015.09.112

Liu, H.J., Watanabe, K., Tojo, S., Sugiyama, T. and Makino, E., 2002. A study on the effect of storage conditions upon rice quality (Part 1) Change in quality of milled rice during storage. Journal of the Japanese Society of Agricultural Machinery.

Makani, M.N., Sargent, S.A., Zotarelli, L., Huber, D.J. and Sims, C.A., 2017. Harvest Interval Has Greater Effect on Periderm Maturity and Storage Quality of Early-maturing, Tablestock Potato than Nitrogen Rate. HortScience: a publication of the American Society for Horticultural Science 52: 1390-1395.

Park, C.E., Kim, Y.S., Park, K.J. and Kim, B.K., 2012. Changes in physicochemical characteristics of rice during storage at different temperatures. Journal of Stored Products Research 48: 25-29.

Patindol, J., Wang, Y.J. and Jane, J.l., 2005. Structure-functionality changes in starch following rough rice storage. Starch-Stärke 57: 197-207.

Ramesh, M., Bhattacharya, K.R. and Mitchell, J.R., 2000. Developments in understanding the basis of cooked-rice texture. Critical Reviews in Food Science and Nutrition 40: 449-460. 10.1080/10408690091189220

Singh, N., Pal, N., Mahajan, G., Singh, S. and Shevkani, K., 2011. Rice grain and starch properties: Effects of nitrogen fertilizer application. Carbohydrate Polymers 86: 219-225. 10.1016/j.carbpol.2011.04.039

Sodhi, N.S., Singh, N., Arora, M. and Singh, J., 2007. Changes in physico-chemical, thermal, cooking and textural properties of rice during aging. Journal of Food Processing & Preservation 27: 387-400.

Tulyathan, V. and Leeharatanaluk, B., 2007. Changes in quality of rice (oryza sativa l.) cv. Khao dawk mali 105 during storage. Journal of Food Biochemistry 31: 415-425.

Unlu, H., Unlu, H.O., Karakurt, Y. and Padem, H., 2010. Organic and conventional production systems, microbial fertilization and plant activators affect tomato quality during storage. African Journal of Biotechnology 9: 7909-7914.

Wang, F., Hu, Q., Mariga, A.M., Cao, C. and Yang, W., 2018. Effect of nano packaging on preservation quality of Nanjing 9108 rice variety at high temperature and humidity. Food Chemistry 239: 23-31. 10.1016/j.foodchem.2017.06.082

Yang, X., Bi, J., Gilbert, R.G., Li, G., Liu, Z., Wang, S. and Ding, Y., 2016. Amylopectin chain length distribution in grains of japonica rice as affected by nitrogen fertilizer and genotype. Journal of Cereal Science 71: 230-238. 10.1016/j.jcs.2016.09.003

Zhang, N., Wen, Y.Y., Yan, S., Mao, H.J., Lei, N.Y., Li, H.Y., Wang, J., Chen, H.T. and Sun, B.G., 2019. The increased stickiness of non-glutinous rice by alkali soaking and its molecular causes. International Journal of Biological Macromolecules 135: 394-399. 10.1016/j.ijbiomac.2019.05.184

Zhou, Z., Robards, K., Helliwell, S. and Blanchard, C., 2002. Ageing of Stored Rice: Changes in Chemical and Physical Attributes. Journal of Cereal Science 35: 65-78.

Zhou, Z., Robards, K., Helliwell, S. and Blanchard, C., 2003. Effect of rice storage on pasting properties of rice flour. Food Research International 36: 625-634.

Zhou, Z., Robards, K., Helliwell, S. and Blanchard, C., 2007. Effect of storage temperature on cooking behaviour of rice. Food Chemistry 105: 491-497.

Zhu, D.W., Zhang, H.C., Guo, B.W., Xu, K., Dai, Q.G., Wei, C.X., Zhou, G.S. and Huo, Z.Y., 2017a. Effects of nitrogen level on structure and physicochemical properties of rice starch. Food Hydrocolloids 63: 525-532. 10.1016/j.foodhyd.2016.09.042

Zhu, D.W., Zhang, H.C., Guo, B.W., Xu, K., Dai, Q.G., Wei, H.Y., Gao, H., Hu, Y.J., Cui, P.Y. and Huo, Z.Y., 2017b. Effects of nitrogen level on yield and quality of japonica soft super rice. Journal of Integrative Agriculture 16: 1018-1027. 10.1016/s2095-3119(16)61577-0

Figure

Click here to access/download;Figure;Fig 1.tif

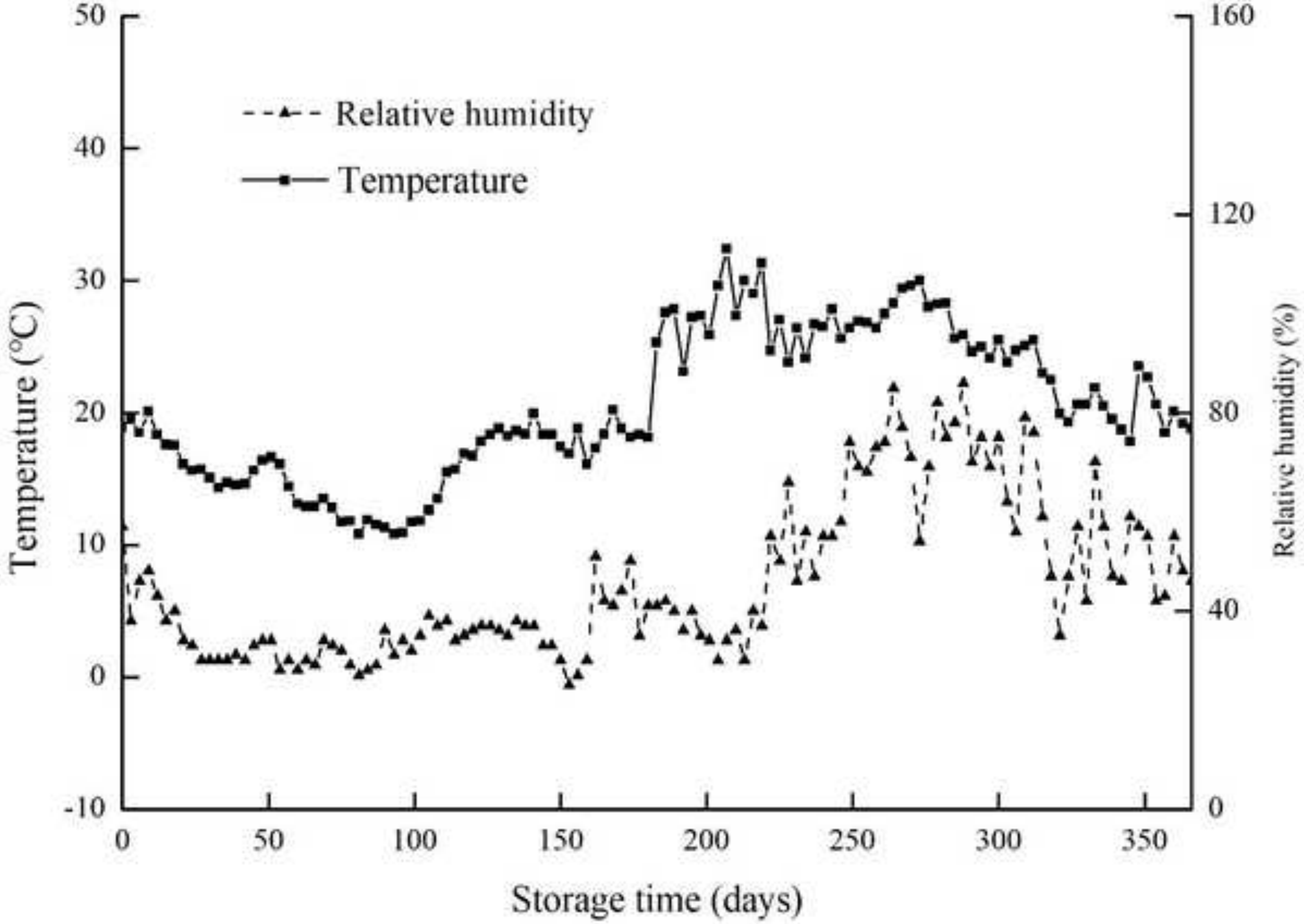

Figure

Click here to access/download;Figure;Fig 2.tif

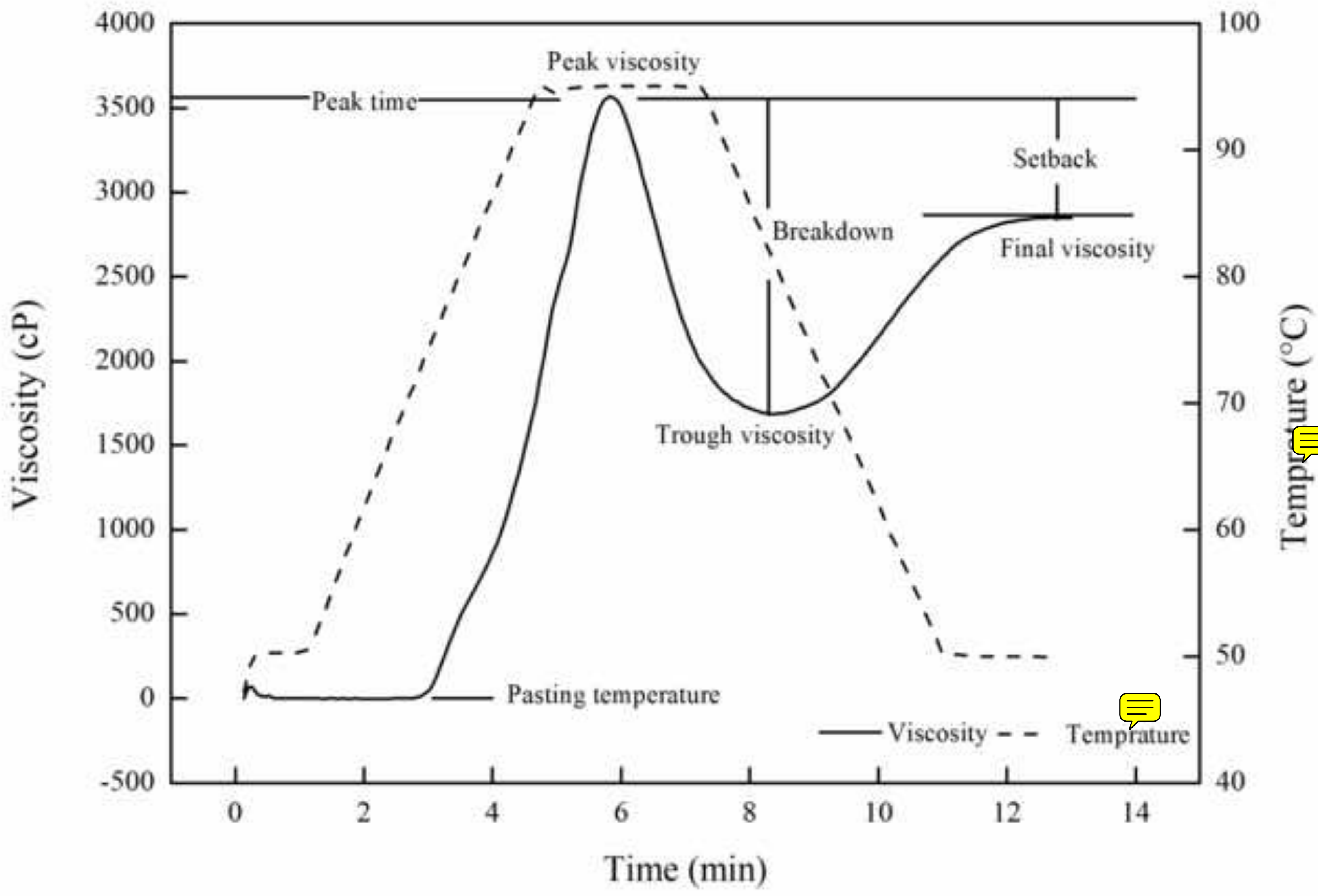

Figure Click here to access/download;Figure;Fig 3.tif

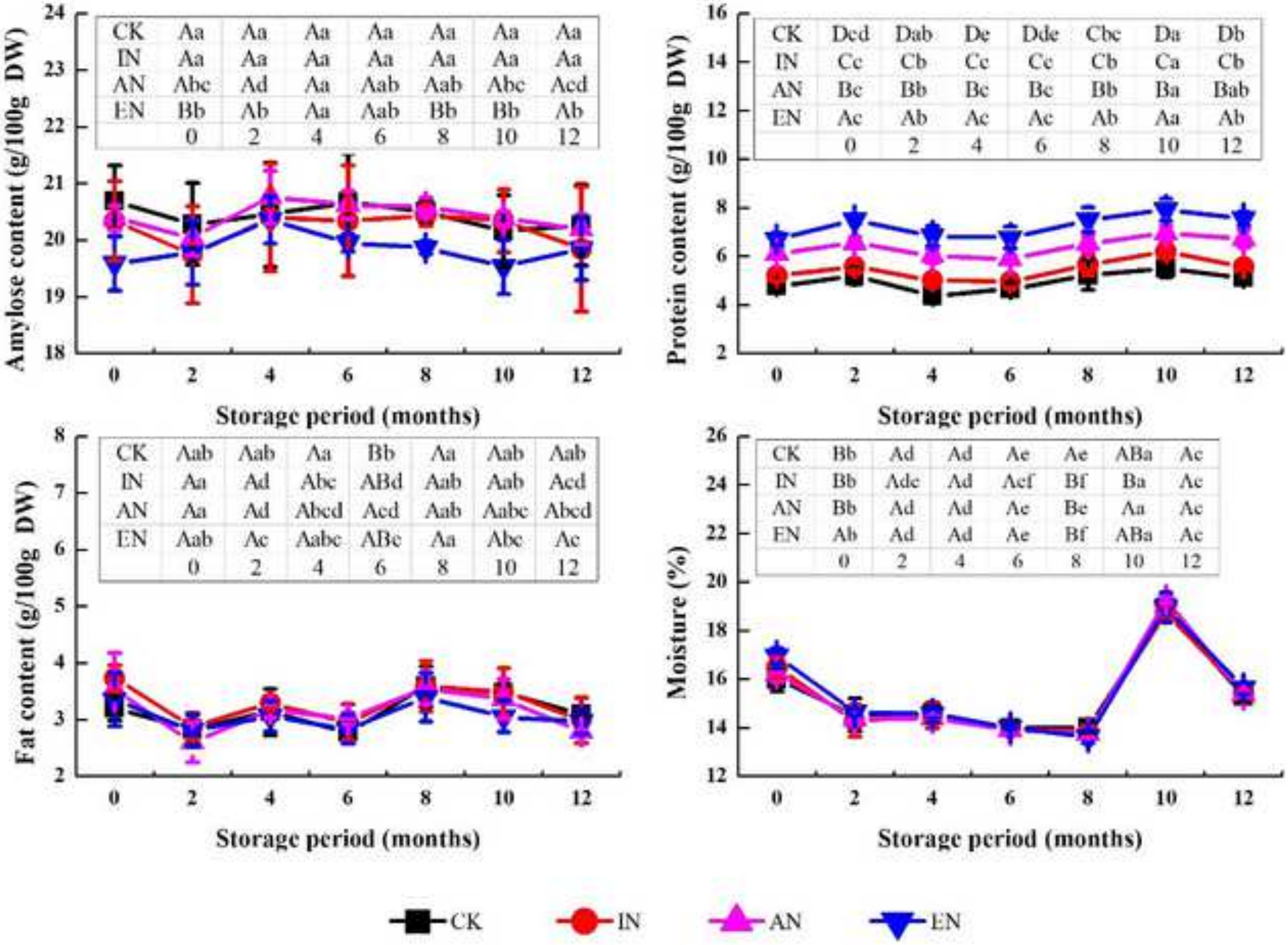

Click here to access/download;Figure;Fig 4.tif

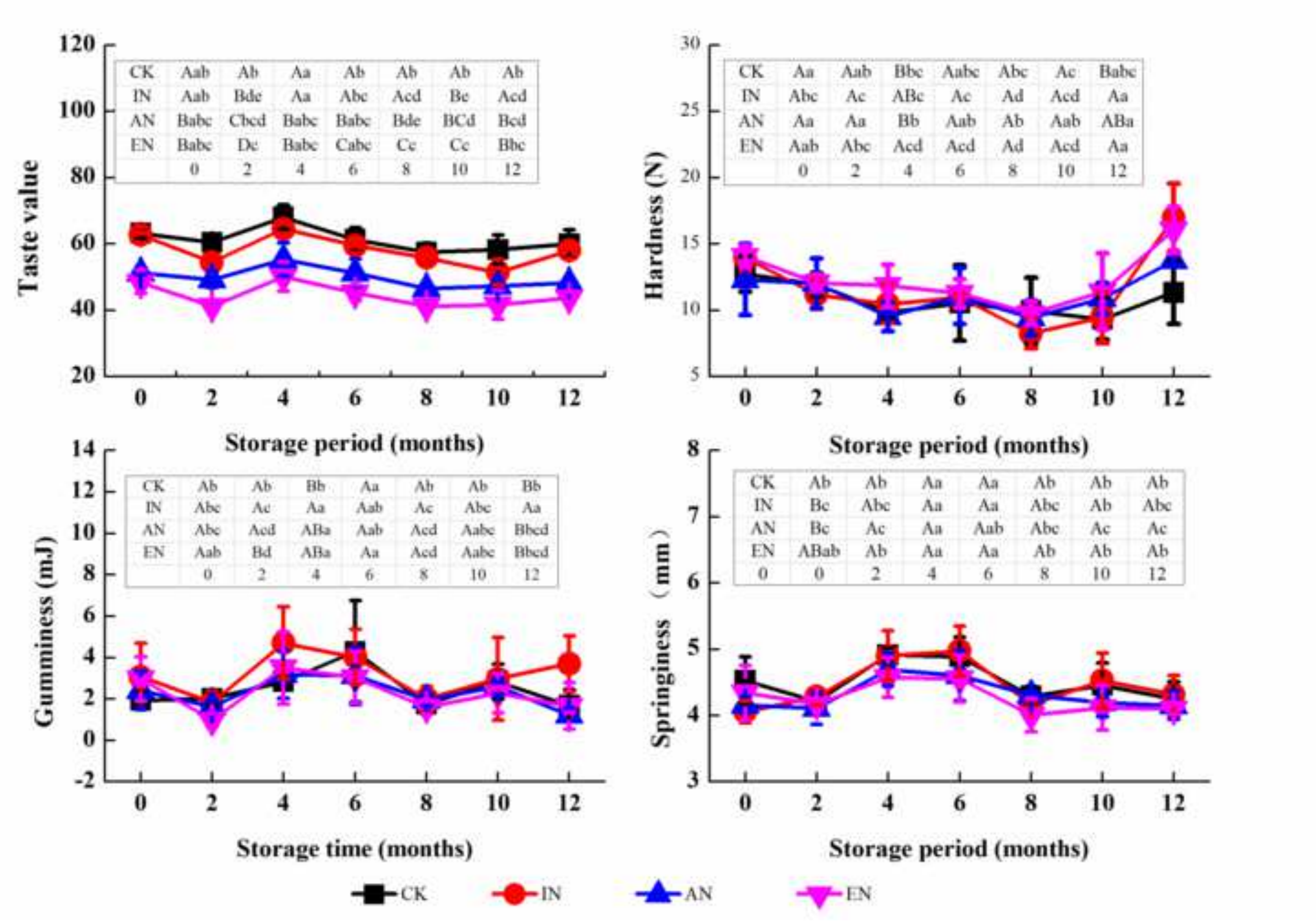

Figure

Click here to access/download;Figure;Fig 5.tif

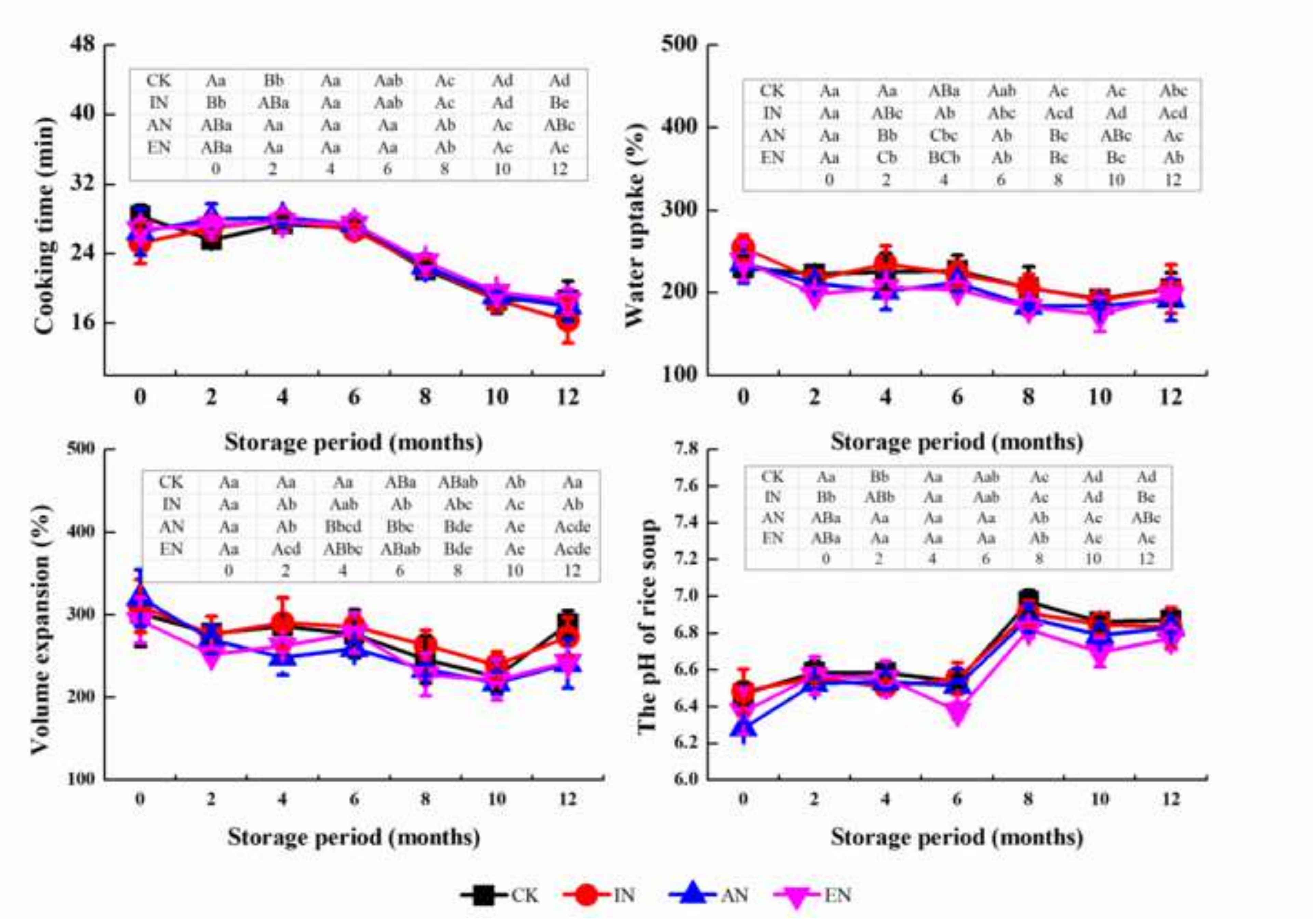

Figure

Click here to access/download;Figure;Fig 6.tif

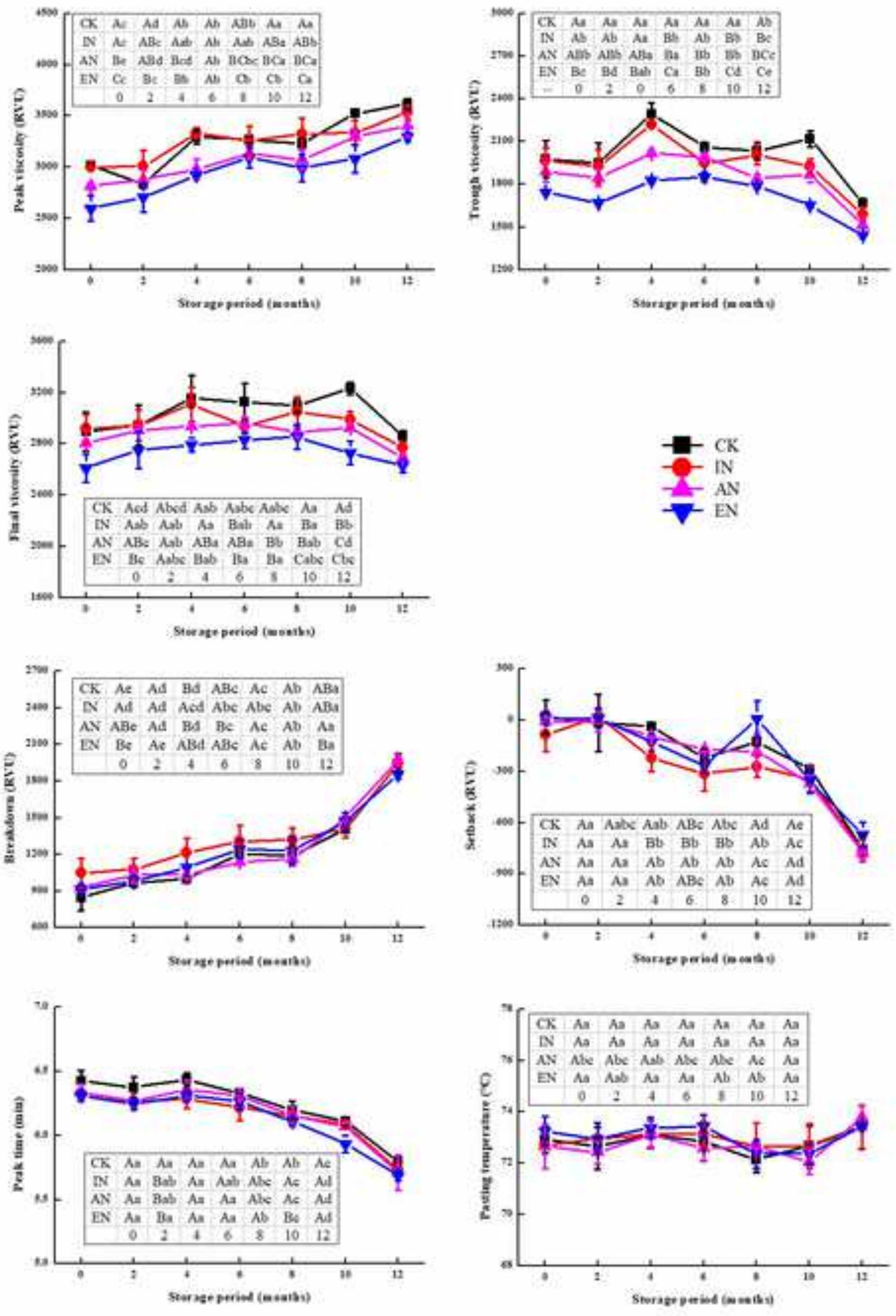

Dear Editor and reviewers,

We wish to express our gratitude to the three reviewers for their professional opinions and suggestions. However, after carefully going through the reviewers' comments, we found that a few questions mentioned by the reviewers were based on the field of agronomy and crop production. Although the present study involves the application of nitrogen in the field, we mainly focused on the differences of eating quality changes during storage due to the different quality of raw materials. Nitrogen fertilizer was applied at different rates only to obtain the raw materials with different qualitative characteristics. The research topic of this paper is pertinent to the field of food science, which may be one of the reasons why the reviewers did not approve of the study. Further, we believe that the research topic of this article is suitable for your journal. Hence, we wish to resubmit the article to your journal, hoping that you will give us another chance to get the article reviewed and published. We also hope that experts from the field of food science will be invited to review the article. Finally, based on the opinions and suggestions of the three reviewers, we have made various modifications to the manuscript. The queries of the three reviewers have been answered as follows:

Reviewer #1: Title: It seems to me that title should changes like as,

1. "Nitrogen fertilizer and storage time impacts on cooking and eating quality of rice".

We thank the reviewer for their suggestion. However, this paper mainly focuses on the effect of nitrogen application rate on the change of eating quality of rice during storage. Nitrogen application is the treatment in the field before rice harvest, and storage time is the treatment condition during storage after rice harvest. If the two phrases are placed parallelly, as in the suggested title, it may not convey our intended meaning. Thus, we think that it will be more appropriate to use the original title.

Abstract:

2. Line 34 & 35: For the first time need use the elaborate terms of CK, IN, AN, EN etc. then use abbreviation in the whole text.

We apologize for our negligence on this issue. We have now changed the sentence as follows："In the present study, the eating and cooking quality of rice treated with 0 (CK), 160 (IN), 260 (AN), and 420 (EN) kg N/ha over 12 months of storage was investigated." has been changed to "The eating and cooking quality (ECQ) of rice treated with 0 (CK, control, 160 (IN, insufficient nitrogen), 260 (AN, adequate nitrogen), and 420 (EN, excessive nitrogen) kg N/ha were analyzed over 12 months of storage." at the Line 25 **&** 27.

3. Line 37: The first words of any sentences need to avoid the use of abbreviation.

We are very sorry for this issue. We have now changed the original sentence to "Results showed that the rate of nitrogen fertilizer application had no significant impact on the taste value after storage. However, EN application significantly increased the hardness ($P<0.05$) and reduced the gumminess of rice ($P<0.05$), and delayed the decline in the viscosity of rice paste by two months." at the Line 27**&** 31.

4. Line 43 & 45: Express the same thing. It is better to compile both of the two sentences.

We have made the correction according to the reviewer's comment, and have deleted some content from the sentences, "During storage, the ratio of protein/amylose showed a significant negative correlation with taste value, springiness, cooking time, and water uptake. In rice with the nitrogen treatments, the ratio of protein/amylose showed a significant negative correlation with peak viscosity, trough viscosity, and setback."

5. Line 47 & 48: Need to re-write. Need to improve the write-up.

We have re-written this part according to the reviewer's suggestion, and have changed the statement "EN had both positive and negative effects on the rice quality during storage. Further investigation is needed to elucidate the mechanism of the changes in rice eating quality during storage." to "It was demonstrated that a rational nitrogen application rate (0–260 kg N/ha) for rice cultivation is particularly important to obtain high ECQ, but EN might be beneficial for the stability of the ECQ during storage." at the Line 32 **&** 34.

6. Keywords: It is better to use only "Nitrogen".

We have changed the phrase from "Nitrogen fertiliser application" to "Nitrogen".

Introduction:

7. If use the spelling fertiliser, then everywhere use the same. But I think it is better to use the spelling "Fertilizer" in the whole text.

We thank the reviewer for their suggestion. We have now changed all instances of the word "fertiliser" to "fertilizer" following American English conventions.

8. Line 59: check the grammar of this sentence.

We are very sorry for the issues associated with language. Since we have now rewritten the whole introduction according to the suggestions by other reviewers, we have deleted the sentence, "the main reasons for this negative effect are the increased protein content and decreased amylose content of rice [2] produced by application of nitrogen."

Materials and methods:

9. Line 82, 83, 86 & 87: Need to re-write these sentences.

We have rewritten this part according to the other reviewer's suggestions, and have changed it to "The following treatment groups were set up for our studies: a control (CK) group without nitrogen treatment (0 kg N/ha), and three treatment groups with insufficient (IN; 160 kg N/ha), adequate (AN; 260 kg N/ha), and excessive (EN; 420 kg N/ha) nitrogen, based on the typical nitrogen fertilization of 260 kg N/ha used by local farmers. Except for the nitrogen application rates, standard rice cultivation procedures were followed as done by the local farmers. The rice variety was Yanfeng 47, which is one of the main local varieties. The seeds were transplanted on May 25, and harvested on October 8 in 2018.". at the Line 77 **&** 87.

Results and discussion:

10. Please check the terms "gumminess", "trough viscosity", "setback" is appropriate for the

rice scientists!!

We thank the reviewer for pointing this out. We have explained the meanings of these terms, and have inserted relevant references. For example, the following statements have been added:

“The texture of the rice cakes (hardness, gumminess, and springiness, defined for instrumental texture analyses in the study by Champagne et al. [1]) was determined with the Texture Analyzer (Brookfield Engineering Laboratories, MA, US) according to Zhang et al. [2].” at the Line 109 **&** 111.

“The pasting parameters of milled rice flour, such as peak viscosity, trough viscosity, breakdown, final viscosity, setback, peak time, and pasting temperature (Fig 2), were determined with a rapid viscosity analyzer (RVA-4, Newport Scientific, Australia).” at the Line 133 **&** 134 and the Line 138 **&** 139.

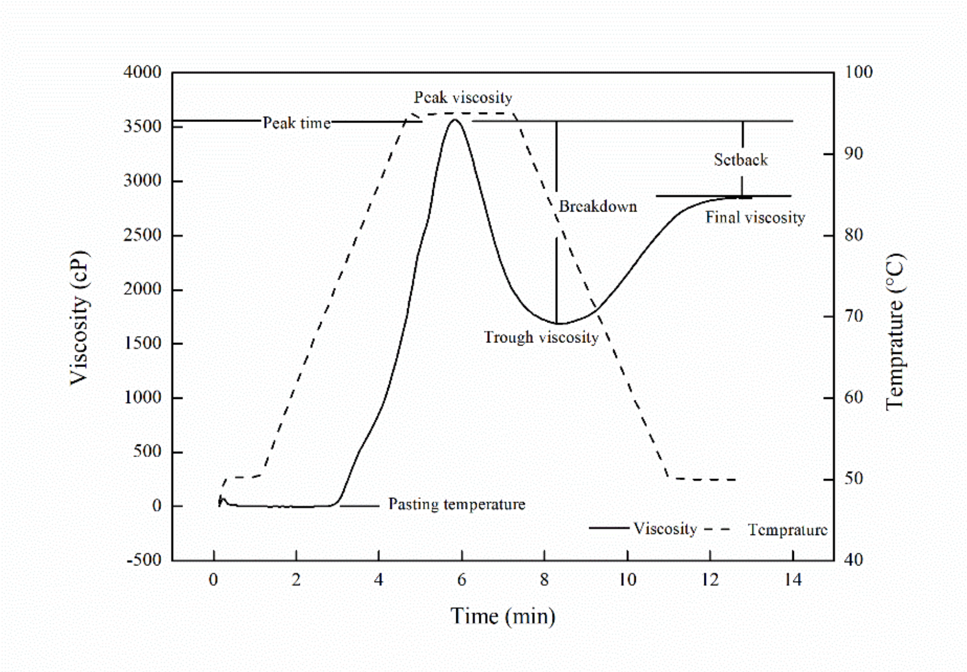

Fig 2. RVA profile of milled rice flour.

11. Line 157 & 158: Delete this table. Because table 1 is not appropriate where as, except 3 all are ns. It is better to express this in a sentence.

Considering the reviewer’s suggestion, we have deleted Table 1, and have added the following sentences to express this: “Among the ECQ indices evaluated, nitrogen application rates and storage time only showed significant interaction effects on gumminess, trough viscosity, and setback ($p < 0.05$; data not shown), indicating that nitrogen application rate has a significant impact the ECQ of rice during postharvest storage. Thus, controlling the nitrogen application rate in the fields is important for maintaining the ECQ of rice during storage.” at the Line 150 **&** 154.

12. Table 2: Need to add the footnote for clarify the terms.

Considering the reviewer's suggestion, we have changed the Table 2 to the Figure 4. And we have now added the footnote to clarify the terms as follows: "CK, control group (0 kg N/hm^2); IN, insufficient nitrogen (160 kg N/hm^2); AN, adequate nitrogen (260 kg N/hm^2); EN, excessive nitrogen (420 kg N/hm^2). For each parameter, the mean values of three replicates ± standard deviation followed by different lowercase letters in the same column differ significantly as per Duncan's test ($P < 0.05$) as a function of storage time. Different uppercase letters in the rows denote significant differences as per Duncan's test ($P < 0.05$) as a function of different nitrogen application rates." the Line 227 **&** 233.

Conclusion:

13. It should be necessary for the expression at a glance for the nitrogen application rate and storage time effect on rice quality. It is also necessary to focus on the output of the whole experiment.

We have now rewritten this part according to the reviewer's suggestion, reorganized the structure, and highlighted the important conclusion for this part as follows: "To our knowledge, this is the first report on the effect of nitrogen fertilizer levels on rice quality during storage. Results showed that excessive nitrogen application significantly increased the hardness, and reduced the gumminess during late storage. AN and EN applications delayed the change in the viscosity of rice paste. In addition, during storage, the protein/amylose ratio showed a better correlation to the ECQ of rice than the protein or amylose contents alone. The results demonstrated that fertilizer application rate affected the quality of the agricultural product during storage by influencing the initial quality of the raw material. Hence, more attention should be paid to the effects of pre-harvest field fertilization on the quality changes of agricultural products during storage, so as to better ensure the high-quality of agricultural products from the field to the plate." at the Line 310 **&** 320.

14. Overall comments: This manuscript English language is poor. It is better to improve the presentation of total English language.

Considering the reviewer's suggestion, the article has been proofread and edited by "Editage" after revision, to address any issues associated with language.

We express our thanks for your helpful comments.

Reviewer #2:

The manuscript presents a research study on the impact of nitrogen application at different doses on rice cooking time and nutritional quality which is/could be an interesting topic to focus on in East and south Asia and sub-Saharan Africa but:

1. The introduction is very poor and does not expose really the problematic, no questions to answer, no assessment of previous studies performed targeting the same subject, no bibliography, etc.

We have now rewritten the introduction according to the reviewer's suggestion, and added statements focusing on the importance, relevant research progress, and existing problems for controlling the deterioration of rice quality during storage. We have also explained how nitrogen application can change the initial quality of rice, which may affect the rice quality during storage, and have added relevant references. The revised statements are as follows at the Line 39 **&** 73 :

"Rice, as a staple food, plays an important role in human diet, and is usually stored for years to meet the needs of people owing to its seasonal growth. However, many studies have reported that the storage process may result in the deterioration of the palatability of rice, including increased hardness and reduced viscosity [3]. Furthermore, the cooking and gelatinization characteristics may be changed, including increase in water absorption, volume expansion rate, cooking time, and setback, as well as a certain decrease in trough viscosity, final viscosity, and breakdown [4]. This deterioration is mostly considered to be related to changes in the interactions between the proteins and starch within the rice grain [5-9].

Numerous efforts have been made in recent years to reduce the deterioration of rice quality due to storage. Low temperature and humidity[10, 11], as well as vacuum or nano packaging [12] have proven to be beneficial in maintaining rice quality during storage. The methods have been applied in the storage of milled rice, which has a high commodity price, but not in rice paddy storage owing to the trade-off between the high cost of the storage method and the relatively low commodity value of rice paddy, which is also the reason why rice paddy is usually stored under natural conditions. To date, no feasible solution to the problem of quality loss during rice paddy storage is available. Except for storage conditions, the initial quality of rice is also a key factor for the end-use quality of rice after storage. Fertilization is important for the establishment of the initial quality of rice, especially the rate of nitrogen application. It is well-documented that nitrogen fertilizers have a prominent effect on the eating and cooking quality (ECQ) of rice [13], mainly due to the increased protein content and decreased amylose content of rice [14] resulting from nitrogen application, which causes an increase in the hardness of rice, decreasing its palatability [15-17], and also alters the rice gelatinization characteristics and cooking quality.

The effect of nitrogen application rate on the quality of fresh rice is well known, but whether this effect persists after a period of storage is still unclear. Previous studies have indicated that fertilization has certain effects on the quality of tomato and potato during storage [18, 19]. However, there are no reports addressing whether different nitrogen fertilization levels affect rice quality during storage. In this study, we explored the dynamic changes in the ECQ after storing rice produced using different nitrogen application rates, and discussed the likely reasons behind the variations in ECQ after storage, with the objective of providing a theoretical basis for the production and quality-control of high-quality rice from the field to the table."

2. The materials and methods section are not well organized;

Nothing was mentioned on the materials, what kind of germplasm (released varieties, cultivars, advanced lines, …)

We apologize for our negligence on this issue. We have now added the relevant statement in the "Materials and methods" section to indicate the rice variety, as follows: "The rice variety was Yanfeng 47, which is one of the main local varieties. The seeds were transplanted on May 25, and harvested on October 8 in 2018." at the Line 85 **&** 87.

3. The different nitrogen fertilizer treatments were adjusted based on the farmer's fertilization rate (which farmer, small or big farmers) and this should not be the case. The treatments should be defined based on the optimal recommended rate (i.e. 0, 50%, 100%, 150%, 200%). As reported in previous studies the economically optimum and ecologically optimum N rates in Asia were estimated to 180–285 kg/ha and 90–150 kg/ha, respectively. Which reference did the authors used and why?

We thank the reviewer for their queries and suggestions. In our opinion, the soil characteristics in different regions within the same province may differ significantly due to the vast area, resulting in different appropriate levels of nitrogen fertilizer for different areas. The field experiment in our study was conducted in Panjin area of the Liaoning Province, located in the Liaohe River Basin, which is one of the main areas for producing rice in China. The nitrogen application rate was based on the local traditional conventional nitrogen application rate of 260 N kg/ha (100%). According to the design principle of threshold experiment, 160 N kg/ha was set as the low nitrogen group (60%), 420 kg N/ha was the high nitrogen group (160%), and no nitrogen treatment acted as the control group (0%). We have added the following statements in the "Materials and methods" section to indicate this: "The following treatment groups were set up for our studies: a control (CK) group without nitrogen treatment (0 kg N/ha), and three treatment groups with insufficient (IN; 160 kg N/ha), adequate (AN; 260 kg N/ha), and excessive (EN; 420 kg N/ha) nitrogen, based on the typical nitrogen fertilization of 260 kg N/ha used by local farmers." at the Line 81 **&** 84.

4. For cooking time normally and the best way to do with a relatively better precise data you should use a mattson cooker.

We express our deepest thanks to the reviewer for their advice. The Mattson cooker is indeed a very good instrument for measuring cooking time. We will consider using this instrument in future experiments. Although we did not use a Mattson cooker to determine the cooking time of rice, our method of estimation was based on a number of previous reports, such as Gujral and Kumar [20], Ziegler et al. [21], and Cui et al. [22]. We also hope to compare our conclusion with other studies.

• Results and discussion

5. Results are not very well presented and structured,

Results presented and the discussion are not balanced. It will better for the authors to present the results and discussion in two separate sections,

We have now rewritten this part according to the reviewer's suggestion. Although we did not divide the results and discussion into two parts, we restructured this part to reorganize the narrative logic of the results, focusing on the integrity of the chain of proof.

6. It could/would be better if the authors could include yield and attributes to the quality traits, this may give more importance to the study

We acknowledge the reviewer’s suggestion. However, we wish to reiterate that in this study, we only assessed the effects of nitrogen fertilization rate during cultivation on the quality of rice following postharvest storage. As the research was mainly conducted from the perspective of food science, we did not include the yield, quality attributes, and other such indicators.

7. Nitrogen application is a determinant practice for seed yield in rice production, how can the authors justify the low seed production under low N? Nowadays, there is more emphasis on high nutritional quality ag. products including high protein content, iron, and zinc. How can the authors justify the low protein content under low N?

We appreciate the reviewer’s concern. Although low nitrogen treatment was unfavorable for rice nutritional quality, such as protein content, low nitrogen treatment was helpful in maintaining the eating quality. Therefore, this study employed a low nitrogen treatment group. As this study mainly focuses on the effect of nitrogen application rate on the quality of rice after storage, the effect of low nitrogen treatment is worth investigating.

8、The manuscript is not well written and structured in the different sections. The study remains of interest but should be completed with some agronomic traits mainly seed yield and attributes. Taking this into consideration the manuscript with the non-innovative results presented cannot be for publication in PLOS ONE journal.

L63: Storage, as one of the main practices of rice after harvest, has a negative (or positive) impact on the ……

L69-70: ………… during storage is called aging which is mostly related to changes in the protein and starch contents of rice and their ………………

L179-184: there is a fig1. caption without the figure

We thank the reviewer for their comment. In order to address the language-related issues mentioned above, we have revised the logical order of the whole introduction. For details, please refer to the response to Comment #1 by the first reviewer.

L179-184 is the annotation for Fig 1. We have now added the image of Fig 1.

However, we did not add the yield and other similar attributes, because this study focuses on the effect of nitrogen application rate on the eating quality of rice after storage, mainly from the perspective of food quality, as stated previously.

Reviewer #3: The title of this manuscript is interesting, and the work can do help to improve rice quality by field management and the storage. Some important data must to be added :

1. The environment of paddy rice growth， the plant time and harvest time， the site of experiment including Longitude and latitude， and the information of soil , especially the nitrogen concentration should be added.

We are grateful to the reviewer for their helpful suggestions. We have now added the following details to the “Materials and methods” section: “The experiment was conducted at Liaohe Delta, Panjin, China (122°14′17″N, 41°9′31″E). The physical and chemical properties of the 0–20 cm soil were as follows: pH 8.2, organic matter 22.57 g/kg, total nitrogen 1.42 g/kg, alkali nitrogen 105.24 mg/kg, available phosphorus 21.61 mg/kg, available potassium 164.22 mg/kg, and bulk density 1.39 g/cm^3.” at the Line 77 **&** 80.

2. The varieties need to introduce, elite rice variety or common rice variety? the planting density is also to be described clearly.

We appreciate the suggestion by the reviewer. We have now added the following details to the “Materials and methods” section: “The rice variety was Yanfeng 47, which is one of the main local varieties. The seeds were transplanted on May 25, and harvested on October 8 in 2018.” at the Line 85 **&** 87.

3. The condition of rice seeds storage is very important in this work, the authour must deribe the storaging condition accuratly and clearly instead of "stored under laboratory conditions".

As suggested by the reviewer, the condition of rice paddy storage was very important in this work. Thus, we have now added the following details in the “Materials and methods” section: “The temperature and humidity conditions in the laboratory were recorded periodically, as shown in Fig 1.

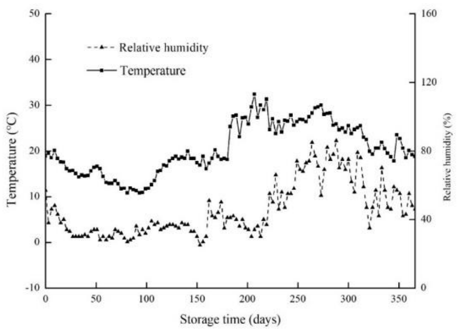

Fig 1. Record of temperature and relative humidity in the laboratory during the experimental period from November 2018 to November 2019.

4. The nitrogen concentration of rice seeds need to be investigated before rice storing.

We appreciate the comment by the reviewer. As this study mainly focuses on the effect of nitrogen application rate on the eating quality of rice following storage, and does not investigate the effect of nitrogen application rate on the concentration of nitrogen in the grain or the nitrogen utilization

rate in rice, we adhered to the problems in the purview of food science. Therefore, the concentration of nitrogen in rice before storage was not determined.

5. Poor discussion and conclusion need to rewrite.

The “Results and discussion” and “Conclusions” sections have been rewritten.

6. Whether Capital letter or lowercase describe in tables and figures?

The capital letters represent the differences between nitrogen fertilizer treatment groups, and lowercase letters represent the differences between the treatment groups at different storage times. We have re-annotated the chart accordingly.

We thank you again for your time, and hope that you will find the article to be modified suitably, and accept it for publication soon.

Sincerely,
Hanling liang

References

1. Champagne ET, Bett KL, Vinyard BT, McClung AM, Barton FE, Moldenhauer K, et al. Correlation between cooked rice texture and rapid visco analyser measurements. Cereal Chem. 1999;76(5):764-71.

2. Zhang N, Wen YY, Yan S, Mao HJ, Lei NY, Li HY, et al. The increased stickiness of non-glutinous rice by alkali soaking and its molecular causes. Int J Biol Macromol. 2019;135:394-9. doi: 10.1016/j.ijbiomac.2019.05.184. PubMed PMID: WOS:000477691600043.

3. National Health and Family Planning Commission of China; National food safety standards. Determination of moisture in food. 2016;GB 5009.3-2016.

4. Keawpeng I, Venkatachalam K. Effect of aging on changes in rice physical qualities. Int Food Res J. 2015;22(6):2180-7. PubMed PMID: WOS:000422964900001.

5. Chrastil J. Protein-starch interactions in rice grains. Influence of storage on oryzenin and starch. J Agric Food Chem. 1990;38(9):1804-9.

6. Patindol J, Wang YJ, Jane JI. Structure-functionality changes in starch following rough rice storage. Starch-Stärke. 2005;57(5):197-207.

7. Huang Y-C, Lai H-M. Characteristics of the starch fine structure and pasting

properties of waxy rice during storage. Food Chem. 2014;152:432-9. doi: 10.1016/j.foodchem.2013.11.144. PubMed PMID: WOS:000332132300060.

8. Guo YB, Cai WR, Tu K, Wang SM, Zhu XL. Key Proteins Causing Changes in Pasting Properties of Rice During Aging. Cereal Chem. 2015;92(4):384-8. doi: 10.1094/cchem-05-14-0104-r. PubMed PMID: WOS:000360818900007.

9. Zhou Z, Robards K, Helliwell S, Blanchard C. Effect of rice storage on pasting properties of rice flour. Food Res Int. 2003;36(6):625-34.

10. Genkawa T, Uchino T, Inoue A, Tanaka F, Hamanaka D. Development of a low-moisture-content storage system for brown rice: Storability at decreased moisture contents. Biosystems Engineering. 2008;99(4):515-22.

11. Park CE, Kim YS, Park KJ, Kim BK. Changes in physicochemical characteristics of rice during storage at different temperatures. Journal of Stored Products Research. 2012;48(48):25-9.

12. Wang F, Hu Q, Mariga AM, Cao C, Yang W. Effect of nano packaging on preservation quality of Nanjing 9108 rice variety at high temperature and humidity. Food Chem. 2018;239:23-31. doi: 10.1016/j.foodchem.2017.06.082. PubMed PMID: WOS:000408740200004.

13. Kaur A, Ghumman A, Singh N, Kaur S, Virdi AS, Riar GS, et al. Effect of different doses of nitrogen on protein profiling, pasting and quality attributes of rice from different cultivars. J Food Sci Technol. 2016;53(5):2452-62. doi: 10.1007/s13197-016-2230-z. PubMed PMID: 27407212; PubMed Central PMCID: PMC4921099.

14. Cao XM, Sun HY, Wang CG, Ren XJ, Liu HF, Zhang ZJ. Effects of late-stage nitrogen fertilizer application on the starch structure and cooking quality of rice. J Sci Food Agric. 2017;98(6):2332-40. Epub 2340. doi: 10.1002/jsfa.8723.

15. Zhu DW, Zhang HC, Guo BW, Xu K, Dai QG, Wei HY, et al. Effects of nitrogen level on yield and quality of japonica soft super rice. J Integr Agric. 2017;16(5):1018-27. doi: 10.1016/s2095-3119(16)61577-0.

16. Singh N, Pal N, Mahajan G, Singh S, Shevkani K. Rice grain and starch properties: Effects of nitrogen fertilizer application. Carbohydr Polym. 2011;86(1):219-25. doi: 10.1016/j.carbpol.2011.04.039.

17. Champagne ET, Bett-Garber KL, Thomson JL, Fitzgerald MA. Unraveling the impact of nitrogen nutrition on cooked rice flavor and texture. Cereal Chem. 2009;86(3):274-

80. doi: 10.1094/CCHEM-86-3-0274.

18. Unlu H, Unlu HO, Karakurt Y, Padem H. Organic and conventional production systems, microbial fertilization and plant activators affect tomato quality during storage. Afr J Biotechnol. 2010;9(46):7909-14. PubMed PMID: WOS:000284481800020.

19. Makani MN, Sargent SA, Zotarelli L, Huber DJ, Sims CA. Harvest Interval Has Greater Effect on Periderm Maturity and Storage Quality of Early-maturing, Tablestock Potato than Nitrogen Rate. HortScience: a publication of the American Society for Horticultural Science. 2017;52(10):1390-5.

20. Gujral HS, Kumar V. Effect of accelerated aging on the physicochemical and textural properties of brown and milled rice. J Food Eng. 2003;59(2-3):117-21.

21. Ziegler V, Ferreira CD, Hoffmann JF, Chaves FC, Vanier NL, De MO, et al. Cooking quality properties and free and bound phenolics content of brown, black, and red rice grains stored at different temperatures for six months. Food Chem. 2018.

22. Cui L, Pan Z, Yue T, Atungulu GG, Berrios J. Effect of Ultrasonic Treatment of Brown Rice at Different Temperatures on Cooking Properties and Quality. Cereal Chem. 2010;87(5):403-8. doi: 10.1094/cchem-02-10-0034.
