# Supplementary material for: Nitrogen fertilizer application rate impacts eating and cooking quality of rice after storage
Source: PLoS One. 2021 Jun 18;16(6):e0253189. doi: 10.1371/journal.pone.0253189 (PMC8213157; doi:10.1371/journal.pone.0253189)
Supplement: Attachment — Submitted filename: Review comments and revision.docx [file pone.0253189.s003.docx]

Dear Editor and reviewers,

We wish to express our gratitude to the three reviewers for their professional opinions and suggestions. However, after carefully going through the reviewers’ comments, we found that a few questions mentioned by the reviewers were based on the field of agronomy and crop production. Although the present study involves the application of nitrogen in the field, we mainly focused on the differences of eating quality changes during storage due to the different quality of raw materials. Nitrogen fertilizer was applied at different rates only to obtain the raw materials with different qualitative characteristics. The research topic of this paper is pertinent to the field of food science, which may be one of the reasons why the reviewers did not approve of the study. Further, we believe that the research topic of this article is suitable for your journal. Hence, we wish to resubmit the article to your journal, hoping that you will give us another chance to get the article reviewed and published. We also hope that experts from the field of food science will be invited to review the article. Finally, based on the opinions and suggestions of the three reviewers, we have made various modifications to the manuscript. The queries of the three reviewers have been answered as follows:

Reviewer #1: Title: It seems to me that title should changes like as,
**1. “Nitrogen fertilizer and storage time impacts on cooking and eating quality of rice”.**
We thank the reviewer for their suggestion. However, this paper mainly focuses on the effect of nitrogen application rate on the change of eating quality of rice during storage. Nitrogen application is the treatment in the field before rice harvest, and storage time is the treatment condition during storage after rice harvest. If the two phrases are placed parallelly, as in the suggested title, it may not convey our intended meaning. Thus, we think that it will be more appropriate to use the original title.

Abstract:
**2. Line 34 & 35: For the first time need use the elaborate terms of CK, IN, AN, EN etc. then use abbreviation in the whole text.**
We apologize for our negligence on this issue. We have now changed the sentence as follows：“In the present study, the eating and cooking quality of rice treated with 0 (CK), 160 (IN), 260 (AN), and 420 (EN) kg N/ha over 12 months of storage was investigated.” has been changed to “The eating and cooking quality (ECQ) of rice treated with 0 (CK, control, 160 (IN, insufficient nitrogen), 260 (AN, adequate nitrogen), and 420 (EN, excessive nitrogen) kg N/ha were analyzed over 12 months of storage.” at the Line 25 **&** 27.

**3. Line 37: The first words of any sentences need to avoid the use of abbreviation.**
We are very sorry for this issue. We have now changed the original sentence to “Results showed that the rate of nitrogen fertilizer application had no significant impact on the taste value after storage. However, EN application significantly increased the hardness (P<0.05) and reduced the gumminess of rice (P<0.05), and delayed the decline in the viscosity of rice paste by two months.” at the Line 27**&** 31.

**4. Line 43 & 45: Express the same thing. It is better to compile both of the two sentences.**
We have made the correction according to the reviewer’s comment, and have deleted some content from the sentences, “During storage, the ratio of protein/amylose showed a significant negative correlation with taste value, springiness, cooking time, and water uptake. In rice with the nitrogen treatments, the ratio of protein/amylose showed a significant negative correlation with peak viscosity, trough viscosity, and setback.”

**5. Line 47 & 48: Need to re-write. Need to improve the write-up.**
We have re-written this part according to the reviewer’s suggestion, and have changed the statement “EN had both positive and negative effects on the rice quality during storage. Further investigation is needed to elucidate the mechanism of the changes in rice eating quality during storage.” to “It was demonstrated that a rational nitrogen application rate (0–260 kg N/ha) for rice cultivation is particularly important to obtain high ECQ, but EN might be beneficial for the stability of the ECQ during storage.” at the Line 32 **&** 34.

**6. Keywords: It is better to use only “Nitrogen”.**
We have changed the phrase from “Nitrogen fertiliser application” to “Nitrogen”.

Introduction:
**7. If use the spelling fertiliser, then everywhere use the same. But I think it is better to use the spelling “Fertilizer” in the whole text.**
We thank the reviewer for their suggestion. We have now changed all instances of the word “fertiliser” to “fertilizer” following American English conventions.

**8. Line 59: check the grammar of this sentence.**
We are very sorry for the issues associated with language. Since we have now rewritten the whole introduction according to the suggestions by other reviewers, we have deleted the sentence, “the main reasons for this negative effect are the increased protein content and decreased amylose content of rice [2] produced by application of nitrogen.”

Materials and methods:
**9. Line 82, 83, 86 & 87: Need to re-write these sentences.**
We have rewritten this part according to the other reviewer’s suggestions, and have changed it to “The following treatment groups were set up for our studies: a control (CK) group without nitrogen treatment (0 kg N/ha), and three treatment groups with insufficient (IN; 160 kg N/ha), adequate (AN; 260 kg N/ha), and excessive (EN; 420 kg N/ha) nitrogen, based on the typical nitrogen fertilization of 260 kg N/ha used by local farmers. Except for the nitrogen application rates, standard rice cultivation procedures were followed as done by the local farmers. The rice variety was Yanfeng 47, which is one of the main local varieties. The seeds were transplanted on May 25, and harvested on October 8 in 2018.”. at the Line 77 **&** 87.

Results and discussion:
**10. Please check the terms “gumminess”, “trough viscosity”, “setback” is appropriate for the rice scientists!!**
We thank the reviewer for pointing this out. We have explained the meanings of these terms, and have inserted relevant references. For example, the following statements have been added:

“The texture of the rice cakes (hardness, gumminess, and springiness, defined for instrumental texture analyses in the study by Champagne et al. [1]) was determined with the Texture Analyzer (Brookfield Engineering Laboratories, MA, US) according to Zhang et al. [2].” at the Line 109 **&** 111.

“The pasting parameters of milled rice flour, such as peak viscosity, trough viscosity, breakdown, final viscosity, setback, peak time, and pasting temperature (Fig 2), were determined with a rapid viscosity analyzer (RVA-4, Newport Scientific, Australia).” at the Line 133 **&** 134 and the Line 138 **&** 139.

**Fig 2. RVA profile of milled rice flour.**

**11. Line 157 & 158: Delete this table. Because table 1 is not appropriate where as, except 3 all are ns. It is better to express this in a sentence.**
Considering the reviewer’s suggestion, we have deleted Table 1, and have added the following sentences to express this: “Among the ECQ indices evaluated, nitrogen application rates and storage time only showed significant interaction effects on gumminess, trough viscosity, and setback (p < 0.05; data not shown), indicating that nitrogen application rate has a significant impact the ECQ of rice during postharvest storage. Thus, controlling the nitrogen application rate in the fields is important for maintaining the ECQ of rice during storage.” at the Line 150 **&** 154.

**12. Table 2: Need to add the footnote for clarify the terms.**
Considering the reviewer’s suggestion, we have changed the Table 2 to the Figure 4. And we have now added the footnote to clarify the terms as follows: “CK, control group (0 kg N/hm^2^); IN, insufficient nitrogen (160 kg N/hm^2^); AN, adequate nitrogen (260 kg N/hm^2^); EN, excessive nitrogen (420 kg N/hm^2^). For each parameter, the mean values of three replicates ± standard deviation followed by different lowercase letters in the same column differ significantly as per Duncan’s test (P < 0.05) as a function of storage time. Different uppercase letters in the rows denote significant differences as per Duncan’s test (P < 0.05) as a function of different nitrogen application rates.” the Line 227 **&** 233.

Conclusion:
**13. It should be necessary for the expression at a glance for the nitrogen application rate and storage time effect on rice quality. It is also necessary to focus on the output of the whole experiment.**

We have now rewritten this part according to the reviewer’s suggestion, reorganized the structure, and highlighted the important conclusion for this part as follows: “To our knowledge, this is the first report on the effect of nitrogen fertilizer levels on rice quality during storage. Results showed that excessive nitrogen application significantly increased the hardness, and reduced the gumminess during late storage. AN and EN applications delayed the change in the viscosity of rice paste. In addition, during storage, the protein/amylose ratio showed a better correlation to the ECQ of rice than the protein or amylose contents alone. The results demonstrated that fertilizer application rate affected the quality of the agricultural product during storage by influencing the initial quality of the raw material. Hence, more attention should be paid to the effects of pre-harvest field fertilization on the quality changes of agricultural products during storage, so as to better ensure the high-quality of agricultural products from the field to the plate.” at the Line 310 **&** 320.

**14. Overall comments: This manuscript English language is poor. It is better to improve the presentation of total English language.**

Considering the reviewer’s suggestion, the article has been proofread and edited by “Editage” after revision, to address any issues associated with language.

We express our thanks for your helpful comments.

Reviewer #2: 
The manuscript presents a research study on the impact of nitrogen application at different doses on rice cooking time and nutritional quality which is/could be an interesting topic to focus on in East and south Asia and sub-Saharan Africa but:

**1. The introduction is very poor and does not expose really the problematic, no questions to answer, no assessment of previous studies performed targeting the same subject, no bibliography, etc.**
We have now rewritten the introduction according to the reviewer’s suggestion, and added statements focusing on the importance, relevant research progress, and existing problems for controlling the deterioration of rice quality during storage. We have also explained how nitrogen application can change the initial quality of rice, which may affect the rice quality during storage, and have added relevant references. The revised statements are as follows at the Line 39 **&** 73 :

“Rice, as a staple food, plays an important role in human diet, and is usually stored for years to meet the needs of people owing to its seasonal growth. However, many studies have reported that the storage process may result in the deterioration of the palatability of rice, including increased hardness and reduced viscosity [3]. Furthermore, the cooking and gelatinization characteristics may be changed, including increase in water absorption, volume expansion rate, cooking time, and setback, as well as a certain decrease in trough viscosity, final viscosity, and breakdown [4]. This deterioration is mostly considered to be related to changes in the interactions between the proteins and starch within the rice grain [5-9].

Numerous efforts have been made in recent years to reduce the deterioration of rice quality due to storage. Low temperature and humidity[10, 11], as well as vacuum or nano packaging [12] have proven to be beneficial in maintaining rice quality during storage. The methods have been applied in the storage of milled rice, which has a high commodity price, but not in rice paddy storage owing to the trade-off between the high cost of the storage method and the relatively low commodity value of rice paddy, which is also the reason why rice paddy is usually stored under natural conditions. To date, no feasible solution to the problem of quality loss during rice paddy storage is available. Except for storage conditions, the initial quality of rice is also a key factor for the end-use quality of rice after storage. Fertilization is important for the establishment of the initial quality of rice, especially the rate of nitrogen application. It is well-documented that nitrogen fertilizers have a prominent effect on the eating and cooking quality (ECQ) of rice [13], mainly due to the increased protein content and decreased amylose content of rice [14] resulting from nitrogen application, which causes an increase in the hardness of rice, decreasing its palatability [15-17], and also alters the rice gelatinization characteristics and cooking quality.

The effect of nitrogen application rate on the quality of fresh rice is well known, but whether this effect persists after a period of storage is still unclear. Previous studies have indicated that fertilization has certain effects on the quality of tomato and potato during storage [18, 19]. However, there are no reports addressing whether different nitrogen fertilization levels affect rice quality during storage. In this study, we explored the dynamic changes in the ECQ after storing rice produced using different nitrogen application rates, and discussed the likely reasons behind the variations in ECQ after storage, with the objective of providing a theoretical basis for the production and quality-control of high-quality rice from the field to the table.”

**2. The materials and methods section are not well organized;
­Nothing was mentioned on the materials, what kind of germplasm (released varieties, cultivars, advanced lines, …)**
­We apologize for our negligence on this issue. We have now added the relevant statement in the “Materials and methods” section to indicate the rice variety, as follows: “The rice variety was Yanfeng 47, which is one of the main local varieties. The seeds were transplanted on May 25, and harvested on October 8 in 2018.” at the Line 85 **&** 87.

**3. The different nitrogen fertilizer treatments were adjusted based on the farmer’s fertilization rate (which farmer, small or big farmers) and this should not be the case. The treatments should be defined based on the optimal recommended rate (i.e. 0, 50%, 100%, 150%, 200%). As reported in previous studies the economically optimum and ecologically optimum N rates in Asia were estimated to 180–285 kg/ha and 90–150 kg/ha, respectively. Which reference did the authors used and why?**
­We thank the reviewer for their queries and suggestions. In our opinion, the soil characteristics in different regions within the same province may differ significantly due to the vast area, resulting in different appropriate levels of nitrogen fertilizer for different areas. The field experiment in our study was conducted in Panjin area of the Liaoning Province, located in the Liaohe River Basin, which is one of the main areas for producing rice in China. The nitrogen application rate was based on the local traditional conventional nitrogen application rate of 260 N kg/ha (100%). According to the design principle of threshold experiment, 160 N kg/ha was set as the low nitrogen group (60%), 420 kg N/ha was the high nitrogen group (160%), and no nitrogen treatment acted as the control group (0%). We have added the following statements in the “Materials and methods” section to indicate this: “The following treatment groups were set up for our studies: a control (CK) group without nitrogen treatment (0 kg N/ha), and three treatment groups with insufficient (IN; 160 kg N/ha), adequate (AN; 260 kg N/ha), and excessive (EN; 420 kg N/ha) nitrogen, based on the typical nitrogen fertilization of 260 kg N/ha used by local farmers.” at the Line 81 **&** 84.

**4. For cooking time normally and the best way to do with a relatively better precise data you should use a mattson cooker.**
We express our deepest thanks to the reviewer for their advice. The Mattson cooker is indeed a very good instrument for measuring cooking time. We will consider using this instrument in future experiments. Although we did not use a Mattson cooker to determine the cooking time of rice, our method of estimation was based on a number of previous reports, such as Gujral and Kumar [20], Ziegler et al. [21], and Cui et al. [22]. We also hope to compare our conclusion with other studies.

• Results and discussion
**­5. Results are not very well presented and structured,
­ Results presented and the discussion are not balanced. It will better for the authors to present the results and discussion in two separate sections,**
­We have now rewritten this part according to the reviewer’s suggestion. Although we did not divide the results and discussion into two parts, we restructured this part to reorganize the narrative logic of the results, focusing on the integrity of the chain of proof.

**6. It could/would be better if the authors could include yield and attributes to the quality traits, this may give more importance to the study**
­We acknowledge the reviewer’s suggestion. However, we wish to reiterate that in this study, we only assessed the effects of nitrogen fertilization rate during cultivation on the quality of rice following postharvest storage. As the research was mainly conducted from the perspective of food science, we did not include the yield, quality attributes, and other such indicators.

**7. Nitrogen application is a determinant practice for seed yield in rice production, how can the authors justify the low seed production under low N? Nowadays, there is more emphasis on high nutritional quality ag. products including high protein content, iron, and zinc. How can the authors justify the low protein content under low N?**
We appreciate the reviewer’s concern. Although low nitrogen treatment was unfavorable for rice nutritional quality, such as protein content, low nitrogen treatment was helpful in maintaining the eating quality. Therefore, this study employed a low nitrogen treatment group. As this study mainly focuses on the effect of nitrogen application rate on the quality of rice after storage, the effect of low nitrogen treatment is worth investigating.

**8、The manuscript is not well written and structured in the different sections. The study remains of interest but should be completed with some agronomic traits mainly seed yield and attributes. Taking this into consideration the manuscript with the non-innovative results presented cannot be for publication in PLOS ONE journal.**

L63: Storage, as one of the main practices of rice after harvest, has a negative (or positive) impact on the ……
L69-70: ………… during storage is called aging which is mostly related to changes in the protein and starch contents of rice and their ………………
L179-184: there is a fig1. caption without the figure

We thank the reviewer for their comment. In order to address the language-related issues mentioned above, we have revised the logical order of the whole introduction. For details, please refer to the response to Comment #1 by the first reviewer.

L179-184 is the annotation for Fig 1. We have now added the image of Fig 1.

However, we did not add the yield and other similar attributes, because this study focuses on the effect of nitrogen application rate on the eating quality of rice after storage, mainly from the perspective of food quality, as stated previously.

Reviewer #3: The title of this manuscript is interesting, and the work can do help to improve rice quality by field management and the storage. Some important data must to be added :
**1. The environment of paddy rice growth，the plant time and harvest time，the site of experiment including Longitude and latitude，and the information of soil , especially the nitrogen concentration should be added.**
We are grateful to the reviewer for their helpful suggestions. We have now added the following details to the “Materials and methods” section: “The experiment was conducted at Liaohe Delta, Panjin, China (122°14′17″N, 41°9′31″E). The physical and chemical properties of the 0–20 cm soil were as follows: pH 8.2, organic matter 22.57 g/kg, total nitrogen 1.42 g/kg, alkali nitrogen 105.24 mg/kg, available phosphorus 21.61 mg/kg, available potassium 164.22 mg/kg, and bulk density 1.39 g/cm^3^.” at the Line 77 **&** 80.

**2. The varieties need to introduce, elite rice variety or common rice variety? the planting density is also to be described clearly.**
We appreciate the suggestion by the reviewer. We have now added the following details to the “Materials and methods” section: “The rice variety was Yanfeng 47, which is one of the main local varieties. The seeds were transplanted on May 25, and harvested on October 8 in 2018.” at the Line 85 **&** 87.

**3. The condition of rice seeds storage is very important in this work, the authour must deribe the storaging condition accuratly and clearly instead of "stored under laboratory conditions".**
As suggested by the reviewer, the condition of rice paddy storage was very important in this work. Thus, we have now added the following details in the “Materials and methods” section: “The temperature and humidity conditions in the laboratory were recorded periodically, as shown in Fig 1.

**Fig 1. Record of temperature and relative humidity in the laboratory during the experimental period from November 2018 to November 2019.**

**4. The nitrogen concentration of rice seeds need to be investigated before rice storing.**
We appreciate the comment by the reviewer. As this study mainly focuses on the effect of nitrogen application rate on the eating quality of rice following storage, and does not investigate the effect of nitrogen application rate on the concentration of nitrogen in the grain or the nitrogen utilization rate in rice, we adhered to the problems in the purview of food science. Therefore, the concentration of nitrogen in rice before storage was not determined.

**5. Poor discussion and conclusion need to rewrite.**

The “Results and discussion” and “Conclusions” sections have been rewritten.

**6. Whether Capital letter or lowercase describe in tables and figures？**

The capital letters represent the differences between nitrogen fertilizer treatment groups, and lowercase letters represent the differences between the treatment groups at different storage times. We have re-annotated the chart accordingly.

We thank you again for your time, and hope that you will find the article to be modified suitably, and accept it for publication soon.

Sincerely,

Hanling liang

References

1.	Champagne ET, Bett KL, Vinyard BT, McClung AM, Barton FE, Moldenhauer K, et al. Correlation between cooked rice texture and rapid visco analyser measurements. Cereal Chem. 1999;76(5):764-71.

2.	Zhang N, Wen YY, Yan S, Mao HJ, Lei NY, Li HY, et al. The increased stickiness of non-glutinous rice by alkali soaking and its molecular causes. Int J Biol Macromol. 2019;135:394-9. doi: 10.1016/j.ijbiomac.2019.05.184. PubMed PMID: WOS:000477691600043.

3.	National Health and Family Planning Commission of China; National food safety standards. Determination of moisture in food. 2016;GB 5009.3-2016.

4.	Keawpeng I, Venkatachalam K. Effect of aging on changes in rice physical qualities. Int Food Res J. 2015;22(6):2180-7. PubMed PMID: WOS:000422964900001.

5.	Chrastil J. Protein-starch interactions in rice grains. Influence of storage on oryzenin and starch. J Agric Food Chem. 1990;38(9):1804-9.

6.	Patindol J, Wang YJ, Jane Jl. Structure‐functionality changes in starch following rough rice storage. Starch‐Stärke. 2005;57(5):197-207.

7.	Huang Y-C, Lai H-M. Characteristics of the starch fine structure and pasting properties of waxy rice during storage. Food Chem. 2014;152:432-9. doi: 10.1016/j.foodchem.2013.11.144. PubMed PMID: WOS:000332132300060.

8.	Guo YB, Cai WR, Tu K, Wang SM, Zhu XL. Key Proteins Causing Changes in Pasting Properties of Rice During Aging. Cereal Chem. 2015;92(4):384-8. doi: 10.1094/cchem-05-14-0104-r. PubMed PMID: WOS:000360818900007.

9.	Zhou Z, Robards K, Helliwell S, Blanchard C. Effect of rice storage on pasting properties of rice flour. Food Res Int. 2003;36(6):625-34.

10.	Genkawa T, Uchino T, Inoue A, Tanaka F, Hamanaka D. Development of a low-moisture-content storage system for brown rice: Storability at decreased moisture contents. Biosystems Engineering. 2008;99(4):515-22.

11.	Park CE, Kim YS, Park KJ, Kim BK. Changes in physicochemical characteristics of rice during storage at different temperatures. Journal of Stored Products Research. 2012;48(48):25-9.

12.	Wang F, Hu Q, Mariga AM, Cao C, Yang W. Effect of nano packaging on preservation quality of Nanjing 9108 rice variety at high temperature and humidity. Food Chem. 2018;239:23-31. doi: 10.1016/j.foodchem.2017.06.082. PubMed PMID: WOS:000408740200004.

13.	Kaur A, Ghumman A, Singh N, Kaur S, Virdi AS, Riar GS, et al. Effect of different doses of nitrogen on protein profiling, pasting and quality attributes of rice from different cultivars. J Food Sci Technol. 2016;53(5):2452-62. doi: 10.1007/s13197-016-2230-z. PubMed PMID: 27407212; PubMed Central PMCID: PMC4921099.

14.	Cao XM, Sun HY, Wang CG, Ren XJ, Liu HF, Zhang ZJ. Effects of late‐stage nitrogen fertilizer application on the starch structure and cooking quality of rice. J Sci Food Agric. 2017;98(6):2332-40. Epub 2340. doi: 10.1002/jsfa.8723.

15.	Zhu DW, Zhang HC, Guo BW, Xu K, Dai QG, Wei HY, et al. Effects of nitrogen level on yield and quality of japonica soft super rice. J Integr Agric. 2017;16(5):1018-27. doi: 10.1016/s2095-3119(16)61577-0.

16.	Singh N, Pal N, Mahajan G, Singh S, Shevkani K. Rice grain and starch properties: Effects of nitrogen fertilizer application. Carbohydr Polym. 2011;86(1):219-25. doi: 10.1016/j.carbpol.2011.04.039.

17.	Champagne ET, Bett‐Garber KL, Thomson JL, Fitzgerald MA. Unraveling the impact of nitrogen nutrition on cooked rice flavor and texture. Cereal Chem. 2009;86(3):274-80. doi: 10.1094/CCHEM-86-3-0274.

18.	Unlu H, Unlu HO, Karakurt Y, Padem H. Organic and conventional production systems, microbial fertilization and plant activators affect tomato quality during storage. Afr J Biotechnol. 2010;9(46):7909-14. PubMed PMID: WOS:000284481800020.

19.	Makani MN, Sargent SA, Zotarelli L, Huber DJ, Sims CA. Harvest Interval Has Greater Effect on Periderm Maturity and Storage Quality of Early-maturing, Tablestock Potato than Nitrogen Rate. HortScience: a publication of the American Society for Horticultural Science. 2017;52(10):1390-5.

20.	Gujral HS, Kumar V. Effect of accelerated aging on the physicochemical and textural properties of brown and milled rice. J Food Eng. 2003;59(2-3):117-21.

21.	Ziegler V, Ferreira CD, Hoffmann JF, Chaves FC, Vanier NL, De MO, et al. Cooking quality properties and free and bound phenolics content of brown, black, and red rice grains stored at different temperatures for six months. Food Chem. 2018.

22.	Cui L, Pan Z, Yue T, Atungulu GG, Berrios J. Effect of Ultrasonic Treatment of Brown Rice at Different Temperatures on Cooking Properties and Quality. Cereal Chem. 2010;87(5):403-8. doi: 10.1094/cchem-02-10-0034.
