# Supplementary material for: Nitrogen fertilizer application rate impacts eating and cooking quality of rice after storage
Source: PLoS One. 2021 Jun 18;16(6):e0253189. doi: 10.1371/journal.pone.0253189 (PMC8213157; doi:10.1371/journal.pone.0253189)
Supplement: Attachment — Submitted filename: Response to Reviewers.docx [file pone.0253189.s005.docx]

Dear Editor and reviewers,

We wish to express our gratitude to the four reviewers for their professional opinions and suggestions. And we have made various modifications to the manuscript as much as we can, based on the opinions and suggestions of the four reviewers. The queries of the four reviewers have been answered as follows:

**Journal Requirements:**

**1. Please ensure that your manuscript meets PLOS ONE's style requirements, including those for file naming.**

Thank you for your valuable comments. We have revised the format of the manuscript according to the relevant documents.

**2. We note that you have included the phrase “data not shown” in your manuscript. Unfortunately, this does not meet our data sharing requirements. PLOS does not permit references to inaccessible data. We require that authors provide all relevant data within the paper, Supporting Information files, or in an acceptable, public repository. Please add a citation to support this phrase or upload the data that corresponds with these findings to a stable repository (such as Figshare or Dryad) and provide and URLs, DOIs, or accession numbers that may be used to access these data. Or, if the data are not a core part of the research being presented in your study, we ask that you remove the phrase that refers to these data.**

Thank you for your suggestion. We have submitted the relevant data as supplementary materials.

**Reviewer #1:**

**The research carried out is correct although very simple. If it is on line with the editorial policy of PLOS ONE, it is ok for me. The minor observations that I have made to this paper are included in the text.**

We appreciate the comment put forth by the reviewer. We have responded to all questions in this article. Please see the text for details.

**Reviewer #2:**

**1. Firstly sample size - where the separate fertiliser treatments grown in separate plots (4 fertiliser treatments times by X number of plots?), or is there more pseudo replication within the experiment one plot/area for each of the fertiliser treatments, with all the ECQ experiments tested on subsamples from the one plot? What area was each N treatment grown over in the field?**

We thank the reviewer for their suggestion. We have added the relevant contents in the section of materials and methods: “*The size of each subplot was 50 m^2^. The transplanting density was 30 cm × 18.2 cm with three seedlings placed in each hill. The experiment was conducted in three replicates*” in lines 81-82.

**2. Secondly, although I know you are concentrating on post-harvest, I feel a few lines need to be added to the discussion regarding the environmental consequences of your findings... Excessive nitrogen is bad for the environment - GHG emissions, N leaching leading to pollution of waterways, eutrophication etc BUT if excessive nitrogen delays quality change in rice, does that reduce the likelihood of food waste?**

We appreciate the suggestion by the reviewer. I have added relevant contents at the end of the discussion: “*Additionally, excessive nitrogen application is detrimental to the environment, such as GHG emissions [**1**], N leaching leading to pollution of waterways, and eutrophication [**2**]. However, we found that excessive nitrogen might be able to delay the aging of rice. This may provide a new evaluation basis of the trade-off between the positive and negative effects in agriculture that occur by excessive nitrogen application.*”, in lines 269-274.

Minor comments

**3. In your rebuttal to previous reviewers you use the phrase "post-harvest storage" I think you should use this phrase more within the manuscript (Abstract, keywords etc) as it conveys the essence of your study better.**

Considering the reviewer’s suggestion, we have revised the relevant content in this article.

**4. line 81: it is standard to present organic matter as a percentage rather than g/kg, please amend**

Thank you for your comments. We have revised the relevant contents as follows: “*The physical and chemical properties of the 0–20-cm soil were as follows: pH 8.2, organic matter 2.26%, total nitrogen 0.14%, alkali nitrogen 10.52%, available phosphorus 0.002%, available potassium 0.016%, and bulk density 1.39 g/cm3.*”, in lines 73-75.

**5. line 87: amend text to "except for the nitrogen application rates, standard practice for rice cultivation procedures were followed by local farmers"**

We are grateful to the reviewer for the helpful suggestions. We have modified it to " *Except for the nitrogen application rates, local farmers applied implemented practice for rice cultivation procedures.*" in lines 79-81.

**6. line 114 and 116: should be author and then year only in brackets - e.g. in the study by Champagne et al. (1999)**

We apologize for our negligence on this issue. We have respected your opinion and modified the reference format of the manuscript according to the PLoS One reference format.

**7. line 193-194: Need to reword - besides, without storage the taste value of rice under EN treatment decreased doesn't make sense.**

Thank you for your opinion. We would like to clarify that compared with the change in taste value of rice before and after one year of storage, the changes were greater between different nitrogen application rates. Therefore, I have revised the original text as follows: “*Rice exposed to different nitrogen application rates showed similar variation trends in taste value during storage (Fig 4). The taste value of rice was significantly reduced with a higher nitrogen application rate and slightly decreased after storage, which is consistent with previous studies [**3**,* *4**]. Moreover, without storage, the taste value of rice under EN treatment significantly decreased from 63 to 48 compared to the CK group. For the CK group, the taste value of rice stored for 12 months slightly decreased from 63 to 60 compared to that observed without storage (Fig 4). Notably, nitrogen application had a greater impact on the taste value of rice than storage under the conditions of this study. Accordingly, controlling the rate of nitrogen application for cultivating rice is particularly important for maintaining the high eating quality of rice during storage.*”, in lines 179-181.

**Reviewer #3:**

The manuscript presented a considerable improvement in the light of the reviewers’ comments; however, there are still some points to consider:

**1. Line 109, 114, 116, 123 : « ….according to Champagne et al. (Champagne et al., 1996) »-« …according to Champagne et al. (1996) »**

Thank you for your suggestion. We have modified the reference format according to the format prescribed by PLoS One.

**2. Line 161: « Nitrogen application did not impact the change of protein, fat, and moisture contents in rice during storage »- This is incorrect for protein content as was clearly reflected by the figure and the statistical analysis. Nitrogen application affects significantly this parameter, whereas, for fat and moisture parameters, there is, in general, no effect of N treatment but a significant effect of storage time. Please verify.**

We apologize for this issue. It is true that nitrogen application significantly affects this parameter, whereas, for fat and moisture parameters, there is, in general, no effect of N treatment except for a significant effect of storage time. However, we focused on the differences in the change trends of those parameters during storage between different nitrogen application rates. We found that the degree change of those parameters during storage was not significantly related to nitrogen application rates. Therefore, we performed a change to the original text, hoping to express our observation more clearly. “*Nitrogen application did not impact the change trend of protein, fat, and moisture contents in rice during storage (Fig 3).*”, in lines 153-154.

**3. Line 168: « …in the study by Gu et al., and was attributed to endogenous degradation (Gu et al., 2019). »-« …in the study by Gu et al. (2019) and was attributed to endogenous degradation. »**

Thank you for your suggestion. The original text has been revised as follows: “*The decline in amylose content of rice during storage was also observed by Gu et al. (2019) [**5**] and was attributed to endogenous degradation.*”, in lines 159-160.

**4. Figures 3, 4, 5: « different lowercase letters in the same column differ significantly as a function of storage time as per Duncan’s test (p< 0.05). Different uppercase letters in the rows denote significant differences as a function of nitrogen application rates… »-It is rather the reverse for column and rows (lowercase for rows and uppercase for column). Please verify.**

Thank you for your careful reading and scrutiny of our manuscript: “*CK, control group (0 kg N/ha); IN, insufficient nitrogen (160 kg N/ha); AN, adequate nitrogen (260 kg N/hm2); EN, excessive nitrogen (420 kg N/ha). Data (mean ± standard deviation, n = 9) with different letters are significantly different (p < 0.05). For each parameter in the inset table, different lowercase letters in the same rows differ significantly as a function of storage time (Duncan’s test; p < 0.05). Different uppercase letters in the column denote significant differences as a function of nitrogen application rates (p < 0.05).*”, in lines 170-175, 218-223, 245-250, 277-282.

**5. For Figs 3, 4, 5 and 6: it is recommended to conserve the same color for the same treatment.**

We thank the reviewer for highlighting this. The color of all figures has been changed.

**6. Line 242: « Except for the pH of the rice soup, there were no significant differences in the changes in cooking quality index during storage between different nitrogen application rates (Fig 5), »- According to the statistical analysis, there is a significant effect for N application in the four parameters in fig 5. Please verify.**

We thank the reviewer for their queries and suggestions. With the prolongation of the storage period, these four indices do have significant changes. However, we focused on the effect of nitrogen application rate on the change range and trend of those indices before and after storage. It can be observed that different nitrogen application rates only have a significant effect on the change trend and range of pH during storage. We changed the original text to: “*Except for the pH of the rice soup, there were no significant differences in the trend in cooking quality index during storage between different nitrogen application rates (Fig 5), which implied that nitrogen fertilizer application during cultivation had little effect on the cooking quality of rice during storage.*”, in lines 226-229.

**7. Line 247: “The changes in the pH of rice soup for CK, IN, AN, and EN groups were -0.7%, +0.7%, -0.3%, and -3%, respectively »- If the comparison concerning the month 0 and the month 12, there is an increase with all the treatments. If it is between 0 and 6 months, verify again especially for AN treatment.**

Thank you for your opinion, which is similar to the opinion expressed in Question 6. As we focused on the changes of pH during storage under different nitrogen treatments, we showed that the differences mainly occurred at 4-6 months. However, we clarified this by the following modifications: “*The pH of rice soup between the four nitrogen treatments had little difference from 0 to 4 months of storage but had significant differences from 4 to 12 months of storage. Moreover, during the entire storage period, EN treatment exacerbated the reduction in the pH of rice soup during storage for 4–6 months. The changes in the pH of rice soup for CK, IN, AN, and EN groups were -0.7%, +0.7%, -0.3%, and -3%, respectively.*”, at lines 230-235.

**8. Line 249: “Further, the EN treatment maintained a low pH of rice soup even after storage of rice for 6 months. »-If the interpretation concerned a specific time (at 6 months), the information was already noted in the line 246. If it is about the total storage time, there is a significant increase in pH from 6 months. Please verify.**

We express our gratitude to the reviewer for the advice. I have revised the original text: “*Further, the EN treatment maintained a lower pH of rice soup even after storage of rice for 6 months compared to other treatments.”*, at lines 234-235.

**9. -Line 309: Fig. 6**

Thank you for your careful reading of my manuscript. We have modified it according to your suggestion, in line 294.

**Reviewer #4:**

Some general comments: The language is not correct and in some part not understandable.

**1. Line 53 to 59: add references.**

Thank you for your valuable comments. Here we have changed to “*Low temperature and humidity [**6**,* *7**], as well as vacuum or nano packaging [**8**], have been proven to be beneficial in maintaining rice quality during storage. Such methods have been applied in milled rice that has a higher commodity price[**9**,* *10**]; however, such methods have not been applied in rice paddy owing to the trade-off between the high cost of those storage methods and the relatively low commodity value of rice paddy. Thus, rice paddy is usually stored under natural conditions[**11**].*” at lines 48-53.

**2. Line 84: In Materials section, you mentioned that all treatments with insufficient (IN; 160 kg 85 N/ha), adequate (AN; 260 kg N/ha), and excessive (EN; 420 kg N/ha) nitrogen, based on the typical nitrogen fertilization of 260 kg N/ha used by local farmers. I think that it is insufficient to specify the group of treatment, you have to add a scientific reference.**

We appreciate the reviewer’s concern. In this study, the treatment of three nitrogen rates is selected according to our previous research results; hence, we have added the following contents in the materials and methods section: “*The following treatment groups were established based on our previous results [**12**] due to their significant effect on the ECQ of rice: a control group without nitrogen treatment (CK; 0 kg N/ha), and three treatment groups with insufficient (IN; 160 kg N/ha), adequate (AN; 260 kg N/ha), and excessive nitrogen (EN; 420 kg N/ha) treatment. The typical nitrogen fertilization of 260 kg N/ha was usually used by local farmers.*”, at lines 75-79.

**3. Line 89: some details in the methods are missing and need to be clarified. In the text, cite the reference number in square brackets as required by the journal.**

We thank the reviewer for their comment. To explain the experimental methods in more detail, the following contents have been added: “*All harvested grains were air-dried for a month to reduce moisture content to approximately 14%; each rice paddy (500 g) was packed in a nylon net bag, placed in a carton, and stored under laboratory conditions for 12 months.*”, at lines 86-88.

**4. Revise the list of references, the instructions on format and style of references are not at all followed.**

Thank you for your suggestion. we have modified the reference format of the full text according to the reference format of PLoS One.

We thank you again for your time and hope that you will find the article to be suitably modified.

Sincerely,

Hanling liang

**References**

1.	Alvaro-Fuentes J, Luis Arrue J, Cantero-Martinez C, Isla R, Plaza-Bonilla D, Quilez D. Fertilization Scenarios in Sprinkler-Irrigated Corn under Mediterranean Conditions: Effects on Greenhouse Gas Emissions. Soil Science Society of America Journal. 2016;80(3):662-71. doi: 10.2136/sssaj2015.04.0156. PubMed PMID: WOS:000378848900012.

2.	Hamonts K, Balaine N, Moltchanova E, Beare M, Thomas S, Wakelin SA, et al. Influence of soil bulk density and matric potential on microbial dynamics, inorganic N transformations, N2O and N-2 fluxes following urea deposition. Soil Biol Biochem. 2013;65:1-11. doi: 10.1016/j.soilbio.2013.05.006. PubMed PMID: WOS:000323686800001.

3.	Zhu DW, Zhang HC, Guo BW, Xu K, Dai QG, Wei HY, et al. Effects of nitrogen level on yield and quality of japonica soft super rice. J Integr Agric. 2017;16(5):1018-27. doi: 10.1016/s2095-3119(16)61577-0.

4.	Liu HJ, Watanabe K, Tojo S, Sugiyama T, Makino E. A study on the effect of storage conditions upon rice quality (Part 1) Change in quality of milled rice during storage. Journal of the Japanese Society of Agricultural Machinery. 2002.

5.	Gu F, Gong B, Gilbert RG, Yu W, Li E, Li C. Relations between changes in starch molecular fine structure and in thermal properties during rice grain storage. Food Chem. 2019;295:484-92. doi: 10.1016/j.foodchem.2019.05.168.

6.	Genkawa T, Uchino T, Inoue A, Tanaka F, Hamanaka D. Development of a low-moisture-content storage system for brown rice: Storability at decreased moisture contents. Biosystems Engineering. 2008;99(4):515-22. doi: 10.1016/j.biosystemseng.2007.12.011.

7.	Park CE, Kim YS, Park KJ, Kim BK. Changes in physicochemical characteristics of rice during storage at different temperatures. Journal of Stored Products Research. 2012;48(48):25-9. doi: 10.1016/j.jspr.2011.08.005.

8.	Wang F, Hu Q, Mariga AM, Cao C, Yang W. Effect of nano packaging on preservation quality of Nanjing 9108 rice variety at high temperature and humidity. Food Chem. 2018;239:23-31. doi: 10.1016/j.foodchem.2017.06.082. PubMed PMID: WOS:000408740200004.

9.	Ahmad U, Alfaro L, Yeboah-Awudzi M, Kyereh E, Dzandu B, Bonilla F, et al. Influence of milling intensity and storage temperature on the quality of Catahoula rice ( Oryza sativa L.). LWT-Food Sci Technol. 2017;75:386-92.

10.	Kim OW. The Quality of Milled Rice with Reference to Whiteness and Packing Conditions during Storage. 2007;14(1):18-23. PubMed PMID: KJD:ART001043654.

11.	Nath S. Indoor Storage of Paddy-Rice in the Lowlands of Papua New Guinea. Ama-Agricultural Mechanization in Asia Africa and Latin America. 2009;40(1):46-9. PubMed PMID: WOS:000266287800009.

12.	Liang HL, Gao SY, Ma JX, Zhang T, Wang TY, Zhang S, et al. Effect of Nitrogen Application Rates on the Nitrogen Utilization, Yield and Quality of Rice. Food and Nutrition Sciences. 2020;12:15. Epub 27. doi: 10.4236/fns.2021.121002
