# Supplementary material for: Nitrogen fertilizer application rate impacts eating and cooking quality of rice after storage
Source: PLoS One. 2021 Jun 18;16(6):e0253189. doi: 10.1371/journal.pone.0253189 (PMC8213157; doi:10.1371/journal.pone.0253189)
Supplement: Attachment — Submitted filename: Response_to_Reviewers.docx [file pone.0253189.s006.docx]

May 25, 2021

*PLOS ONE*

Dear Editor and Reviewers,

We wish to re-submit the manuscript titled “Nitrogen fertilizer application rate impacts eating and cooking quality of rice after storage.” The manuscript ID is PONE-D-21-06634R1.

We wish to express our gratitude to the three reviewers for their professional opinions and suggestions. We have made various modifications to the manuscript to the best of our ability based on the opinions and suggestions of the three reviewers. The queries of the three reviewers have been answered below.

We thank you again for your time and hope that you will find the article to be suitably modified.

Sincerely,

Hanling Liang

College of Food Science, Shenyang Agricultural University, Shenyang 110866, People’s Republic of China

**Journal Requirements:**

**Please review your reference list to ensure that it is complete and correct. If you have cited papers that have been retracted, please include the rationale for doing so in the manuscript text, or remove these references and replace them with relevant current references. Any changes to the reference list should be mentioned in the rebuttal letter that accompanies your revised manuscript. If you need to cite a retracted article, indicate the article’s retracted status in the References list and also include a citation and full reference for the retraction notice.**
Response: Thank you for your careful reading and scrutiny of our manuscript. We have reviewed the reference list and ensured that it is complete and correct. We have added three references as mentioned below in the response to the 2^nd^ comment from Reviewer 4 and removed a reference as mentioned in response to the 4^th^ comment from Reviewer 4.

**Comments to the author**
Reviewer’s responses to questions

Reviewer #1:

**The paper can now be accepted for publication since the authors have modified their previous text according to my corrections.**

Response: We appreciate the comment put forth by the reviewer.

Reviewer #3:

**1. “The decline in amylose content of rice during storage was also observed by Gu et al. (2019) [5] and was attributed to endogenous degradation”
-Delete the year and parentheses**

Response: Thank you for your comments. We have deleted the relevant content.

**2. The reference number does not match that in the revised manuscript. Verify.
-Insert the revised reference list in the manuscript and verify again all the correspondence author-number.**

Response: We are grateful to the reviewer for the helpful suggestions. We have verified all the reference numbers within the manuscript and checked the correspondence between authors and numbers.

Reviewer #4:

**1. Line 42: rewrite this sentence, so long**

Response: Thank you for your comments. We have revised the relevant content as follows (lines 40–42):

*However, several studies have reported that the storage process may result in the deterioration of the eating quality of the rice, such as increased hardness and reduced viscosity [**1**].*

**2. Line 50-53: rewrite this sentence, not clear**

Response: We thank the reviewer for their suggestion. We have rewritten the relevant content as follows and added references 11–13 for clarification (lines 49–53):

*Such methods have been applied in milled rice that has a higher commodity price [11, 12]; however, such methods have not been widely applied in the storage of paddy rice, owing to the trade-off between the high cost of those storage methods and the relatively low commodity value of paddy rice. Thus, paddy rice is usually stored under natural conditions [**13**].*

**3. Line 153-167: add more percentages to describe the results**

Response: Thank you for your comments. We have revised the relevant content as follows (lines 155–163):

*Nitrogen application did not impact the change trend of protein, fat, and moisture contents in rice during storage (Fig 3). The change in rice content before and after storage under the four nitrogen application treatments had a range of 8%–12% for protein, 5%–11% for fat, and 29%–30% for moisture. Amylose content in the CK and IN samples did not vary significantly during storage; however, EN treatment significantly exacerbated the rate of amylose decline during the period of storage, spanning from 4 to 10 months. During the storage period of 4–10 months, the amylose content decreased by 1% in the CK samples and 4% with EN treatment, which is approximately four times that of CK. This finding demonstrated that nitrogen application in the field changed the starch metabolism of rice during storage.*

**4. Line 394: revise this reference.**

Response: Thank you for your careful reading and scrutiny of our manuscript. This reference describes our previous research results that we published in *Food and Nutrition Sciences*. Because the journal is not included in SCI, it may not be found on Web of Science and other channels, but the article can be retrieved through Google Scholar or by the DOI. Here, in order to avoid misunderstanding, I deleted this reference and made relevant modifications in the materials and methods as follows (lines 75–82):

*The following treatment groups were established based on our previous research, in which the effect of nitrogen application rates (0, 160, 210, 260, 315, and 420 kg N/ha) on rice quality was investigated, and due to significant effects on the ECQ of rice: a control group without nitrogen treatment (CK; 0 kg N/ha) and three treatment groups with insufficient (IN; 160 kg N/ha), adequate (AN; 260 kg N/ha), and excessive nitrogen (EN; 420 kg N/ha) treatment. The typical nitrogen fertilization of 260 kg N/ha was usually used by local farmers. Except for the nitrogen application rates, standard practices for rice cultivation were followed by local farmers.*

**5. Line 397: some reference, delete it. 22. National food safety standards. Determination of fat in food. Beijing, China:National Health and Family Planning Commission; 2016.
23. National food safety standards. Determination of moisture in food. Beijing, China:
National Health and Family Planning Commission; 2016.**

Response: Thank you for your comments. We have deleted the relevant content.
